# Population genetic structure, linkage disequilibrium and effective population size of conserved and extensively raised village chicken populations of Southern Africa

**DOI:** 10.3389/fgene.2015.00013

**Published:** 2015-02-03

**Authors:** Khulekani S. Khanyile, Edgar F. Dzomba, Farai C. Muchadeyi

**Affiliations:** ^1^Biotechnology Platform, Agricultural Research CouncilPretoria, South Africa; ^2^Discipline of Genetics, School of Life Sciences, University of KwaZulu-NatalPietermaritzburg, South Africa

**Keywords:** genetic diversity, village chickens, SNPs, linkage disequilibrium, effective population size

## Abstract

Extensively raised village chickens are considered a valuable source of biodiversity, with genetic variability developed over thousands of years that ought to be characterized and utilized. Surveys that can reveal a population's genetic structure and provide an insight into its demographic history will give valuable information that can be used to manage and conserve important indigenous animal genetic resources. This study reports population diversity and structure, linkage disequilibrium and effective population sizes of Southern African village chickens and conservation flocks from South Africa. DNA samples from 312 chickens from South African village and conservation flocks (*n* = 146), Malawi (*n* = 30) and Zimbabwe (*n* = 136) were genotyped using the Illumina iSelect chicken SNP60K BeadChip. Population genetic structure analysis distinguished the four conservation flocks from the village chicken populations. Of the four flocks, the Ovambo clustered closer to the village chickens particularly those sampled from South Africa. Clustering of the village chickens followed a geographic gradient whereby South African chickens were closer to those from Zimbabwe than to chickens from Malawi. Different conservation flocks seemed to have maintained different components of the ancestral genomes with a higher proportion of village chicken diversity found in the Ovambo population. Overall population LD averaged over chromosomes ranged from 0.03 ± 0.07 to 0.58 ± 0.41 and averaged 0.15 ± 0.16. Higher LD, ranging from 0.29 to 0.36, was observed between SNP markers that were less than 10 kb apart in the conservation flocks. LD in the conservation flocks steadily decreased to 0.15 (PK) and 0.24 (VD) at SNP marker interval of 500 kb. Genomewide LD decay in the village chickens from Malawi, Zimbabwe and South Africa followed a similar trend as the conservation flocks although the mean LD values for the investigated SNP intervals were lower. The results suggest low effective population sizes particularly in the conservation flocks. The utility and limitations of the iselect chicken SNP60K in village chicken populations is discussed.

## Introduction

Extensively raised village chickens are considered a valuable source of biodiversity, with genetic variability developed over thousands of years, that could be useful in future for improvement in response to climate change and consumer demands (Delany, 2004). This diversity ought to be characterized, conserved and manipulated to suit production systems such as free-range organic farming. Surveys that can reveal the effective population sizes, inbreeding levels, the effects of natural and artificial selection, as well as population bottleneck events that shaped these populations' current genetic structures will provide valuable information that can be used to manage and conserve these indigenous animal genetic resources. Previous diversity studies (Muchadeyi et al., 2007; Mtileni et al., 2010, 2011b) used microsatellite markers that were of sparse density and could not be used to extensively estimate the population demographic parameters. However, in the presence of dense marker sets, advanced statistical genomics methods can now be used to build an understanding of population genetic and demographic parameters in the absence of pedigree records.

Linkage disequilibrium (LD), defined as non-random association of alleles at two or more loci (Hedrick, 2004; Qanbari et al., 2010) is a useful tool in genetics and evolutionary biology. Its patterns are useful in understanding the levels of inbreeding (García-Gámez et al., 2012) the genetic background of animal populations (Porto-Neto et al., 2014) and assists in the fine mapping of genes and quantitative trait loci (QTL) of economically important traits (Wragg et al., 2012). The decay and extent of LD at a pair-wise distance can be used to determine the evolutionary history of populations (Andreescu et al., 2007; Lu et al., 2012; Wragg et al., 2012). LD will therefore be of use particularly in extensively raised chicken populations in smallholder farming systems where it can be used to calculate population genetic parameters in the absence of pedigree data.

The advent of whole genome sequencing and high density SNP genotyping technologies has resulted in increased marker density and facilitated estimation of LD in a number of domesticated animals including chickens. The completion of the first draft of the chicken genome (Hillier et al., 2004) made it possible for the development of high density markers (Groenen et al., 2011; Kranis et al., 2013). The Illumina iSelect chicken SNP60K BeadChip consists of a panel of 57,636 SNPs (Groenen et al., 2011) that have found utility in population genetic studies and LD analysis in various commercial (Qanbari et al., 2010) and traditional chicken populations (Wragg et al., 2012) as well as in other analyses such as mapping of Mendelian traits (Wragg et al., 2012) and in copy number variation screening (Jia et al., 2012).

This study sought to investigate the underlying population diversity and structure, and the extent and decay of LD in extensively raised village chicken populations of Southern Africa using samples obtained from South Africa (SA), Malawi (Mal), and Zimbabwe (Zim). These chicken populations are raised by smallholder communal farmers under village chicken farming systems characterized by low input management, uncontrolled mating systems and intermixing of flocks within and between villages (Muchadeyi et al., 2007). Population genetic structure of these chickens could be a function of small flock sizes, inbreeding (since farmers retain breeding stock from within flocks over a number of generations), as well natural selection from disease outbreaks, extreme weather conditions and poor quality feed. The objectives of the study were therefore to (i) investigate the population structure and diversity (ii) investigate the extent and decay of LD and (iii) estimate LD-based effective population sizes of extensively raised chickens from Southern Africa (Zimbabwe, Malawi, and South Africa) and provide baseline information for their management and conservation purposes.

## Materials and methods

### Chickens populations, blood collection and SNP genotyping

**Figure 1:**
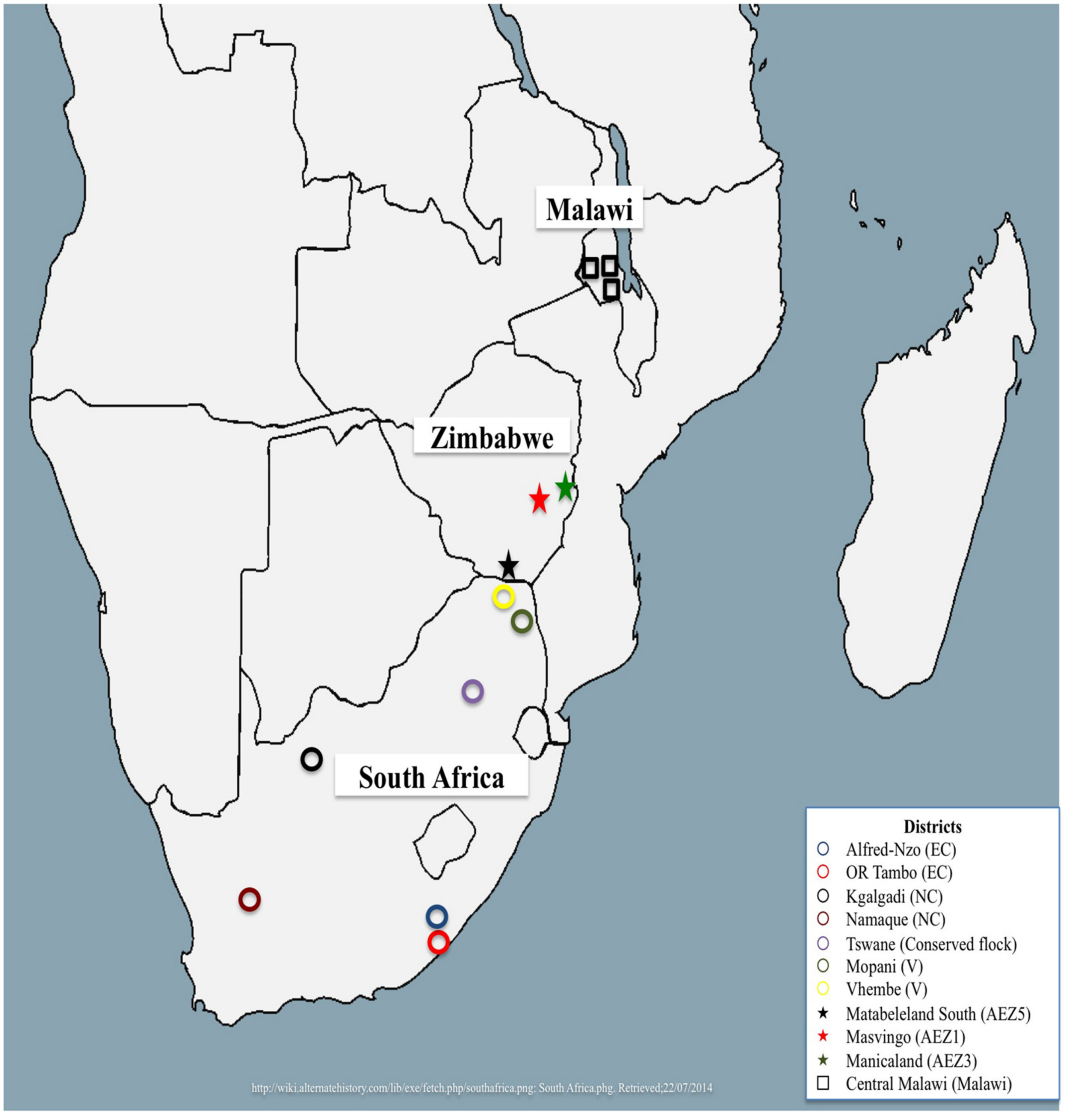
**Map showing sampled regions from Malawi, South Africa, and Zimbabwe**.

A total of 312 village chickens were randomly sampled from South Africa, Malawi, and Zimbabwe. South African village chickens were represented by chickens from Limpopo (*n* = 15), Eastern Cape (*n* = 26) and Northern Cape (*n* = 35) provinces, and four conserved flocks of Venda (VD, *n* = 20), Naked Neck (NN, *n* = 20), Potchefstroom Koekoek (PK, *n* = 20) and Ovambo (OV, *n* = 10) that are kept at the Agriculture Research Council Poultry Breeding Resource at Irene in Pretoria. Detailed sampling of these populations was described by Mtileni et al. (2011b). A total of 135 village chickens were sampled from three Zimbabwean agro-ecological zones (AEZ) of AEZ1 (*n* = 92), AEZ3 (*n* = 34), and AEZ5 (*n* = 10). The detailed sampling of Zimbabwe chicken populations is described by Muchadeyi et al. (2007). The sampling locations for both the conservation flocks and field populations of South Africa and Zimbabwe are indicated in Figure 1. Thirty chickens sampled from one region of central Malawi (Figure 1) were also used in the study. Basically the study selected individuals, households, villages, and regions to obtain genetically unrelated individuals representing a wide geographical location. The distances between villages within a district ranged from 20 to 40 km, and 100 to 500 km between districts within a province and over 1000 km between provinces. The number of individuals varied from 2 to 10 per village depending on per household chicken density in each village. All the village chickens used in this study were not selected for any commercial production traits and were raised by communal farmers under a scavenging system of production.

Blood samples had been collected on FTA Micro Cards (Whatman Bio Science, UK) described in the previous studies (Muchadeyi et al., 2007; Mtileni et al., 2011b). DNA was extracted from these FTA cards using a modified Qiagen® DNeasy Blood and Tissue protocol. DNA quality was checked on a 1% agarose gel where bright sharp bands where observed indicating an intact DNA (no degradation) and DNA concentration of 50 ng/μl for each sample was used for genotyping.

### SNP genotypes and data preparation

SNP genotyping was done using the Illumina chicken iSelect SNP60 Bead chip using the Infinium assay compatible with the Illumina HiScan SQ genotsyping platform at the Agricultural Research Council-Biotechnology Platform in South Africa. This Inifinium whole genome genotyping assay is designed to interrogate a large number of SNPs at unlimited levels of loci multiplexing (www.illumina.com). SNP calling was done using Illumina Genome Studio v2.0. The genotype input file was converted into a PLINK (v1.07) (Purcell et al., 2007) input file using a plug-in compatible with the Genome Studio program. SNP quality control was done in a number of stages depending on the downstream analysis.

### Basic population genetic parameters

A single data set consisting of all seven populations was filtered for SNPs that were monomorphic or had minor allele frequency (MAF) ≤0.02 and this resulted in a total sample of 311 chickens across the seven populations. There were 54,115 SNPs available to estimate observed and expected heterozygosity indices (H_O_ and H_E_) as well as the inbreeding co-efficient of each population using PLINK (v1.07) software (Purcell et al., 2007). The inbreeding coefficients of the populations were tested for deviation from zero using paired *t*-tests of the Proc *t*-Test in Statistical Analysis System (SAS, 2011). PLINK (v1.07) software was also used to measure minor allele frequency distribution per population using the comprehensive data set before pruning for MAF. Bins were set for minor allele frequencies of 0–0.05, 0.05–0.1, 0.1–0.2, 0.2–0.3, 0.3–0.4, and 0.4–0.5. The proportion of SNPs per bin was calculated by dividing the number of markers per bin by the total number of markers included in the MAF estimation.

### Population structure

A comprehensive SNP data set with all seven populations was filtered to remove SNPs that were either on sex chromosomes or had their positions unmapped. Markers with missing data >5%; that were monomorphic or had a MAF ≤2% were removed. Individuals with missing genotypes of more than 5% were also dropped. Closely related individuals, as inferred by a kinship estimate ≥0.45, were filtered out of the data set together with SNPs in high linkage disequilibrium at a threshold of LD ≥0.2. As a result 29,942 SNPs from 266 village chickens were available for analyses.

A principal component analysis (PCA) was then performed to illustrate the relationship among the extensively raised chicken populations using the Golden Helix SNP Variation Suit (SVS) version 8.1 (Golden Helix Inc., 2014).

In addition, ADMIXTURE 1.23 software (Alexander et al., 2009) was used to infer the most propable number of ancestral populations based on the SNP genotype data. Prior information on breed of origin was not used in the determination of the distinct genetic populations or in assigning individuals to populations. Admixture was run from *K* = 2 to *K* = 8 and the optimal number of clusters (*K*-value) was determined as that which had the lowest cross validation error (CV-error).

### Linkage disequilibrium

SNP data for the individual populations were quality-controlled in order to remove SNPs (i) on sex chromosomes or those there were not mapped, (ii) with MAF ≤5%, (iii) those that deviated from Hardy-Weinberg equilibrium (HWE) (*P* ≤ 0.001), (iv) with missing genotypes (>5%) as well as for individual chickens with missing genotypes (>5%) and high kinship (IBD ≥0.45) using PLINK (v1.07) (Purcell et al., 2007). After filtering, 46,973, 48,359, 48,482 as well as 38,976, 42,858, 44,920, and 44,403 SNPs on 28 autosomal chromosomes were available for the Malawian (*n* = 29), Zimbabwean (*n* = 121) and South African field populations (*n* = 62) and conservation flocks of NN (*n* = 15), OV (*n* = 9), VD (*n* = 12), and PK (*n* = 18), respectively. The level of identity by descent in the resultant data sets were 0.022, 0.038, 0.060, and 0.072 for the PK, OV, NN, and VD conservation flocks and 0.002, 0.006, and 0.004 for the South African, Malawian, and Zimbabwean village chickens, respectively. These individual population data sets were used for the estimation of linkage disequilibrium and associated estimates.

A pair-wise *r*^2^ estimation was used to measure LD between pairs of SNPs within a chromosome and population using PLINK (v1.07) program (Purcell et al., 2007) for SNPs on autosomal chromosomes 1–28 that had passed the quality control as described above. The *r*^2^ measure, which is defined as the squared correlation coefficient of alleles at two loci was chosen because it is independent of allele frequency (Lu et al., 2012). Briefly, its calculation, considers two loci, *A* and *B*, each locus having two alleles (denoted *A_1_, A_2_*; *B_1_, B_2_*, respectively) (Qanbari et al., 2010). The frequencies of the haplotypes will then be denoted as *f_11_, f_12_, f_21_*, and *f_22_* for haplotypes *A_1_B_1_, A_1_B_2_, A_2_B_1_*, and *A_2_B_2_*, respectively and as f*_A1_*, f*_A2_* f*_B1_*, and f*_B2_* for A_1_, A_2_, B_1_, and B_2_, respectively. From this, *r*^2^ was then be calculated as:

(1) $$r^{2}=\frac{{\left(f_{11}f_{22}-f_{12}f_{21}\right)}^{2}}{fA_{1}fA_{2}fB_{1}fB_{2}}.$$

By default, PLINK only reports *r*^2^-values above 0.2 and to allow reporting of all *r*^2^-values observed in the populations, the –*r*^2^*–window-ld 0* option was used. An additional option, *–r2 –window-snp 5000 –kb 10000*, allowed for estimation of *r*^2^ for SNP marker pairs separated by at most 5000 SNPs and within a 10 MB SNP interval.

An Analysis of Variance (ANOVA) was conducted using the Generalized Linear Model procedure (Proc GLM) in the SAS (2011) to determine the effects of chromosome, population, the interaction of chromosome-by-population, and SNP marker interval (bp) on LD using the following model:

(2) $$r^{2}ij=\;\mu +{\text{Pop}}_{i}+{\text{Gga}}_{j}+{\left(\text{Pop}\times \text{Gga}\right)}_{ij}+b{\text{SNP}}_{\text{int}}+e_{ik},$$

where: *r*^2^_*ij*_ was the pairwise LD; μ was the overall population mean and Pop_i_ was the effect of the *i*th chicken population from Malawi, Zimbabwe or South Africa; Gga*_j_* was the effect of the *j*th chromosome 1–28; and SNP_int_ represented the effects of SNP interval which were defined as the distance between markers (number of base pairs) and fitted as a covariate with regression coefficient *b*. The *F*-test from the ANOVA analysis was used to determine the significance of factors included in the model at *P* ≤ 0.05. Linkage disequilibrium decay was estimated genomewide for all subpopulations. Sliding window bins for LD decay were set at 10, 20, 40, 60, 100, 200, 500, 1000, 2000, and 5000 kb for chromosomes 1–28. An additional analysis of the macro-chromosomes 1–5 was done with bins up to 10,000 kb.

### Trends in effective population size

The relationship between *Ne*, recombination frequency and expected LD (*r*^2^) was determined using the following equation from Corbin et al. (2012);

(3) $$E\left[r_{adj}^{2}\right]={\left(\alpha +4N_{e}c\right)}^{-1}$$

where α = 1 when assuming no mutations and 2 if mutation was considered,

(4) $$r_{adj}^{2}=r^{2}-\frac{1}{2n},\;c$$

was the recombination rate, and *n* was the chromosomal sample size. The effective population size *Ne*, as

(5) $$\frac{1}{2c}$$

generations, was estimated from the adjusted *r*^2^_*adj*_ values related to a given genetic distance *d* in Morgans, assuming, *c* = *d* (Qanbari et al., 2010).

For each pair of SNPs on each chromosome, recombination rate was estimated by converting physical marker interval length *x_i_*(MB) to the corresponding genetic length *c_i_* using the formula: *c*_*i*_ = ō_*i*_x_*i*_, where ō*_i_* is the average ratio of Morgans per kilo base pair on chromosome *i*, which was taken from the physical lengths of the chicken genome v74 (Ensembl, 2013). The genetic length of chromosomes was adopted from Hillier et al. (2004). The *r*^2^-values range between 0 and 1, whereby a zero value indicates uncorrelated SNPs while a value of one reflects SNPs that are perfectly correlated (Qanbari et al., 2010).

The trends in effective population sizes for each of the defined subpopulations were then estimated by setting bins at 10, 20, 40, 60, 100, 200, 500, 1000, 2000, and 5000 kb. The bins were designed to cover the genome in tens, hundreds, thousands, and hundred thousand base pairs.

## Results

### SNP marker characteristics

Minor allele frequency averaged 0.29 (Table 1) and over 8.5% of the SNPs on the Illumina iSelect chicken SNP60K panel had a MAF of less than 0.05 (Supplementary Figure 1). An analysis of the distribution of MAF across all populations showed that over 10% of the markers were within the 0–10% MAF threshold. Of the 57,636 SNPs on the panel, 29,942 were used for the determination of population structure and diversity whilst a range of 38,976 (in the Ovambo) to 48,482 (in Zimbabwe) were used for estimating LD in the different populations (Table 1). Majority of the SNPs excluded were either monomorphic or had minor allele frequencies ≤0.02 and were therefore considered not informative for the populations. Over 1000 SNPs had missing genotypes amongst the seven populations. SNPs located on unknown chromosomes, linkage groups, and sex chromosomes were also excluded from further analysis. The proportion of SNPs used for further analysis was 51% for the whole population for estimation of population structure and was over 80% for the village chickens from Malawi, South Africa and Zimbabwe and ranged from 67 to 77% in the conservation flocks for the estimation of LD.

**Table 1:** **SNP distribution after quality control and the minor allele frequency (MAF), observed (H_O_), expected (H_E_) heterozygosities and inbreeding coefficient (F) of Malawi, South African field (SAField), Zimbabwean chicken populations as well as the Naked Neck (NN), Potchefstroom Koekoek (PK), Ovambo (OV) and Venda (VD) conservation flocks from South Africa**.

	**Malawi**	**Zimbabwe**	**SAField**	**NN**	**PK**	**OV**	**VD**
**SNPs**							
Total	57,636	57,636	57,636	57,636	57,636	57,636	57,636
Sex chromosome	3202	3202	3202	3202	3202	3202	3202
Unmapped	172	172	172	172	172	172	172
Monomorphic	1992	1878	1501	4488	2341	4702	2975
MAF ≤0.02	3706	2409	2047	9648	5191	4317	5204
HWE (*P* ≥ 0.001)	207	278	193	102	109	101	77
Missing Gen > 0.05	1384	1338	2039	1048	2218	2284	1086
**PARAMETERS**							
H_O_	0.62 ± 0.00	0.62 ± 0.03	0.62 ± 0.00	0.62 ± 0.00	0.62 ± 0.00	0.62 ± 0.00	0.62 ± 0.00
H_E_	0.68 ± 0.02	0.66 ± 0.04	0.65 ± 0.05	0.70 ± 0.14	0.68 ± 0.06	0.65 ± 0.01	0.71 ± 0.00
Mean MAF ±SD	0.27 ± 0.14	0.28 ± 0.13	0.29 ± 0.13	0.26 ± 0.14	0.26 ± 0.14	0.28 ± 0.13	0.24 ± 0.14
F^*^	0.15 ± 0.07	0.12 ± 0.12	0.07 ± 0.14	0.21 ± 0.03	0.14 ± 0.15	0.07 ± 0.04	0.24 ± 0.18

* *Inbreeding coefficients of all populations were significantly > 0 at P ≤ 0.05*.

### Basic population genetic parameters

Observed heterozygosity values averaged 0.62 ± 0.003 across all seven populations. Overall, H_0_ in all populations was lower than expected (0.67 ± 0.048) and the populations were therefore significantly inbred (*P* ≤ 0.05) inbred. Heterozygosity estimates and inbreeding coefficients were high in the conservation flocks compared to the village flocks. Of the conservation flocks, Ovambo chickens had the lowest levels of inbreeding.

### Population structure using PCA and admixture analysis

**Figure 2:**
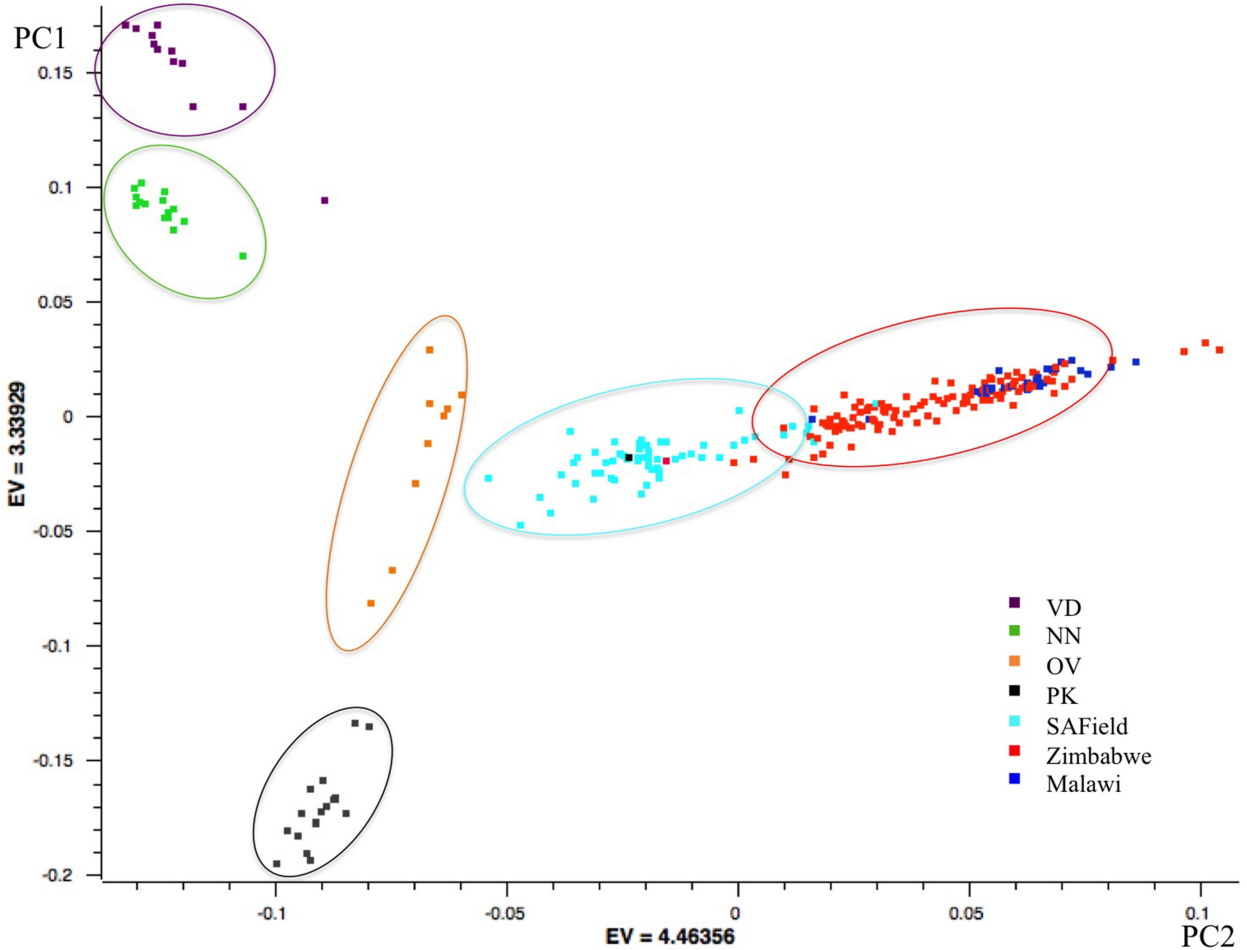
**PCA based clustering of populations**. Population clusters are within the ovals and the color of each oval represents the predominant chicken population. VD, Venda conservation flocks; NN, Naked Neck conservation flocks; OV, Ovambo conservation flocks; PK, Potchefstroom Koekoek conservation flock; SAField, village chickens from South Africa; Zimbabwe, village chickens from Zimbabwe; Malawi, village chickens from Malawi.

Results of the first principal component showed the conserved Venda, Ovambo, Naked Neck and Potchefstroom Koekoek chickens from South Africa grouped into four distinct clusters separated from the village chicken populations. Of the four flocks, the Ovambo clustered closer to the village chickens particularly those sampled from South Africa. Clustering of the village chickens followed a geographic gradient whereby South African chickens were closer to those from Zimbabwe than to chickens from Malawi. The chickens from Malawi clustered together with some chickens from Zimbabwe (Figure 2).

**Figure 3:**
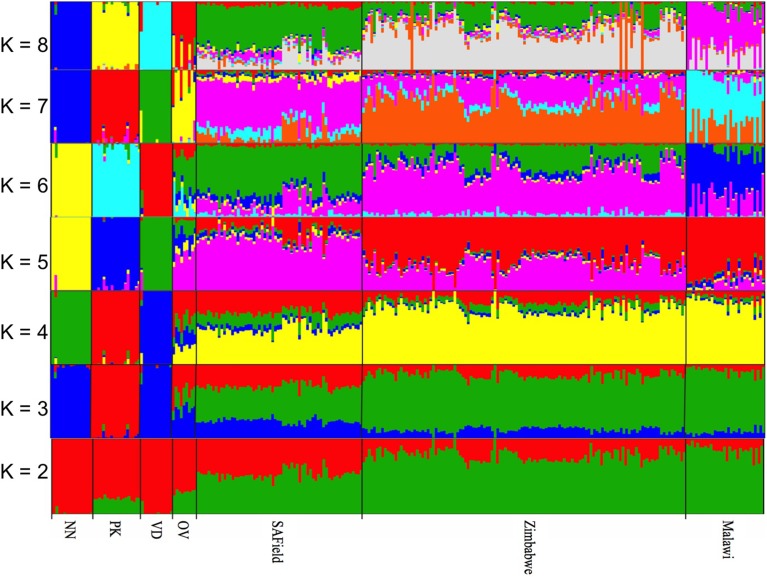
**Population substructuring using ADMIXTURE**. VD, Venda conservation flocks; NN, Naked Neck conservation flocks; OV, Ovambo conservation flocks; PK, Potchefstroom Koekoek conservation flock; SAField, village chickens from South Africa; Zimbabwe, village chickens from Zimbabwe; Malawi, village chickens from Malawi.

The optimal *K*-value for admixture was *K* = 6 (Supplementary Figure 2) corresponding to the conserved (i) Naked Neck, (ii) Potchefstroom Koekoek (iii) Venda (iv) Ovambo and (v) the village chickens from Malawi and (vi) village chicken from South Africa and Zimbabwe that had slight variations in allele frequencies (Figure 3). Similar to PCA results, the Ovambo chickens clustered separately but with greater diversity and some similarity to the village chicken populations (Figure 3). The clustering also revealed conservation of some village chickens' ancestral genomic components in the Naked Neck and Potchefstroom Koekoek flocks. The Ovambo had diverse genomic elements with some that were concentrated in the Potchefstroom Koekoek, Naked Neck and Venda conservation flocks and others that were found in the village chicken populations from the three countries.

### LD estimates and the effects of chromosome, SNP intervals and breed

For each chromosome, total length, number of SNPs and average SNP interval are shown in Supplementary Table 1. Table 2 summarizes *r*^2^ values for the 28 autosomal chromosomes for the four conservation flocks and three village chicken populations of Southern Africa. The SNP interval was not consistent across the genome, ranging from a distance of 0.01 to 0.1 Mb. Macro-chromosomes showed the highest marker distance followed by intermediate chromosomes. SNP intervals were shorter for the micro-chromosomes (Supplementary Table 1). Number of SNPs per chromosome varied with chromosome size between the macro (chromosomes 1–5) that had the highest number of SNPs (976 to 3443) and micro (chromosome 16–28) with fewer numbers of SNPs (5 to 832) per chromosome.

**Table 2:** **Linkage disequilibrium of Malawi, Zimbabwe, South African Field (SAField) village chickens and the Naked Neck (NN), Venda (VD), Potchefstroom Koekoek (PK) and Ovambo (OV) conservation flocks**.

	**Linkage Disequilibrium (*r*^2^**)						
	**Malawi**	**Zimbabwe**	**SAField**	**NN**	**VD**	**PK**	**OV**
1	0.09±0.14	0.05±0.10	0.05±0.10	0.23±0.26	0.26±0.28	0.17±0.21	0.21±0.23
2	0.9±0.14	0.05±0.10	0.06±0.11	0.22±0.25	0.27±0.29	0.17±0.21	0.21±0.24
3	0.09±0.14	0.05±0.09	0.05±0.09	0.24±0.26	0.26±0.28	0.17±0.21	0.20±0.22
4	0.09±0.14	0.05±0.10	0.06±0.10	0.23±0.26	0.26±0.29	0.16±0.20	0.21±0.23
5	0.08±0.13	0.05±0.09	0.06±0.10	0.23±0.27	0.24±0.26	0.16±0.20	0.21±0.22
6	0.08±0.12	0.04±0.08	0.05±0.08	0.24±0.26	0.25±0.26	0.17±0.21	0.22±0.23
7	0.09±0.14	0.05±0.10	0.05±0.10	0.23±0.26	0.26±0.29	0.16±0.20	0.20±0.22
8	0.11±0.17	0.07±0.15	0.07±0.15	0.23±0.26	0.28±0.29	0.21±0.26	0.22±0.25
9	0.08±0.12	0.04±0.08	0.05±0.08	0.19±0.22	0.22±0.26	0.16±0.20	0.20±0.22
10	0.08±0.12	0.04±0.09	0.05±0.09	0.21±0.24	0.28±0.31	0.18±0.22	0.20±0.20
11	0.9±0.14	0.06±0.11	0.06±0.12	0.28±0.29	0.22±0.33	0.23±0.26	0.20±0.23
12	0.09±0.12	0.05±0.09	0.05±0.09	0.24±0.27	0.33±0.33	0.17±0.21	0.22±0.23
13	0.09±0.13	0.05±0.09	0.05±0.09	0.23±0.26	0.26±0.28	0.18±0.22	0.22±0.23
14	0.10±0.15	0.05±0.10	0.05±0.10	0.22±0.25	0.24±0.28	0.17±0.21	0.21±0.23
15	0.10±0.15	0.07±0.12	0.07±0.12	0.25±0.27	0.26±0.28	0.18±0.23	0.23±0.24
16	0.10±0.15	0.04±0.08	0.12±0.14	0.27±0.28	0.58±0.41	0.23±0.28	0.25±0.27
17	0.09±0.14	0.05±0.11	0.06±0.11	0.24±0.27	0.24±0.27	0.18±0.22	0.22±0.24
18	0.08±0.11	0.04±0.08	0.04±0.07	0.23±0.26	0.22±0.26	0.17±0.21	0.20±0.22
19	0.08±0.11	0.04±0.09	0.04±0.09	0.21±0.23	0.22±0.25	0.17±0.22	0.19±0.21
20	0.09±0.14	0.05±0.10	0.06±0.11	0.22±0.26	0.32±0.31	0.21±0.25	0.19±0.21
21	0.09±0.13	0.05±0.09	0.05±0.09	0.21±0.25	0.30±0.30	0.18±0.22	0.19±0.21
22	0.08±0.13	0.05±0.09	0.05±0.11	0.21±0.25	0.25±0.29	0.16±0.21	0.19±0.22
23	0.08±0.11	0.03±0.07	0.04±0.08	0.27±0.29	0.23±0.27	0.16±0.21	0.19±0.22
24	0.08±0.11	0.04±0.09	0.04±0.09	0.20±0.21	0.24±0.27	0.14±0.18	0.21±0.22
25	0.07±0.14	0.03±0.07	0.04±0.07	0.17±0.21	0.17±0.22	0.15±0.20	0.19±0.21
26	0.07±0.11	0.04±0.07	0.04±0.07	0.23±0.26	0.20±0.23	0.17±0.21	0.18±0.19
27	0.09±0.14	0.04±0.10	0.05±0.09	0.22±0.25	0.21±0.26	0.17±0.21	0.21±0.23
28	0.09±0.13	0.05±0.10	0.05±0.10	0.19±0.22	0.25±0.29	0.16±0.21	0.18±0.21

Overall population LD averaged over chromosomes ranged from 0.03 ± 0.07 to 0.58 ± 0.41 and averaged 0.15 ± 0.16 (Table 2). The *F*-test results from the analysis of variance showed that pairwise LD varied significantly (*P* < 0.001) among chromosomes, populations and their interaction as well as with SNP marker interval (Table 3). Chromosome 16 had high LD in all the conservation flocks, and in Malawi and South Africa, whilst chromosome 25 had low LD across all populations. Chickens from Malawi had higher LD compared to those from South Africa and Zimbabwe. The conservation flocks had significantly higher LD compared to the village flocks. The Naked Neck and Venda conservation flocks had higher LD across chromosomes compared to Potchefstroom Koekoek and Ovambo chickens. The highest LD (0.58 ± 0.41) was observed in the Venda conservation flocks.

**Table 3:** **The effects of population, chromosome and SNP marker interval on linkage disequilibrium (*r^2^*)**.

**Factor**	**DF**	**SS**	**MS**	***F*-value**	***P*-value**
Population	6	4363.4	727.23	1,892,555	^***^
Chromosome	27	629.76	23.32	606.98	^***^
SNP Distance	1	954.77	954.77	24,846.4	^***^
Population x chromosome	162	734.08	4.53	117.92	^***^

*** *p < 0.0001*.

**Figure 4:**
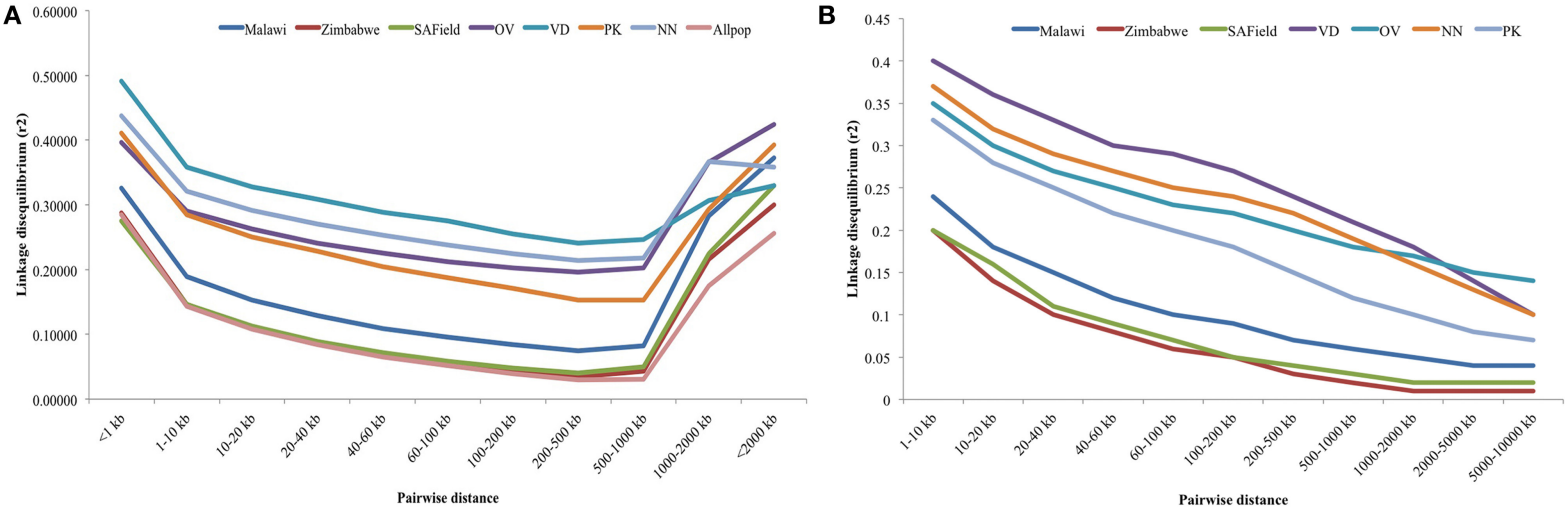
**(A)** Average LD decay with increased physical distance between SNPs for chromosomes 1–28. VD, Venda conservation flocks; NN, Naked Neck conservation flocks; OV, Ovambo conservation flocks; PK, Potchefstroom Koekoek conservation flock; SAField, village chickens from South Africa; Zimbabwe, village chickens from Zimbabwe; Malawi, village chickens from Malawi. **(B)** Average LD decay with increased physical distance between SNPs for macro-chromosomes 1–5. VD, Venda conservation flocks; NN, Naked Neck conservation flocks; OV, Ovambo conservation flocks; PK, Potchefstroom Koekoek conservation flock; SAField, village chickens from South Africa; Zimbabwe, village chickens from Zimbabwe; Malawi, village chickens from Malawi.

Linkage disequilibrium depended on SNP distance. Plots of the rate of LD decay over marker distance over all 28 autosomes and for macro-chromosomes 1–5 are given in Figures 4A,B, respectively. Higher LD, ranging from 0.29 to 0.36, was observed between SNP markers that were less than 10 kb apart in the conservation flocks. Within this window, LD was highest in the Venda (*LD* = 0.36) followed by Naked Neck (>0.33) and least in Ovambo and Potchefstroom Koekoek (0.29). LD in the conservation flocks steadily decreased to 0.15 (PK) and 0.24 (VD) at SNP marker interval of 500 kbp. A sudden increase in LD was observed at 500 kbp SNP interval. Genomewide LD decay in the village chickens from Malawi, Zimbabwe and South Africa followed a similar trend as the conservation flocks but the mean LD values at different SNP intervals were lower (Figure 4A).

An additional analysis of LD decay of the macro-chromosomes 1–5 was performed for all the populations and results are illustrated in Figure 4B. LD for these macro-chromosomes was high (>0.3) for the conservation flocks for markers within 10 kb intervals and steadily decreased to values lower than 0.15 beyond 8 Mb SNP intervals. Lower LDs of 0.2 were observed in the village chickens at 10 kb SNP interval and then decreased to less than 0.05 beyond 8 Mb.

The trends observed for LD decay per chromosome per population were similar to those for the overall genomewide population (Supplementary Figure 3). However, distinctly high LD was observed for chromosome 16 particularly for the Venda Conservation flock. The sudden increase in LD at SNP intervals higher than 500 kb was observed in some micro-chromosomes (7–11, 13, 14, 20, 23, 24, 26–28) (Supplementary Figure 2). This trend was observed only in certain conservation flocks on chromosomes 9 (VD; OV; NN), 10 (PK; VD; OV; NN), 13 and 14 (VD), and 24 (PK).

### Effective population size over the past generations

**Figure 5:**
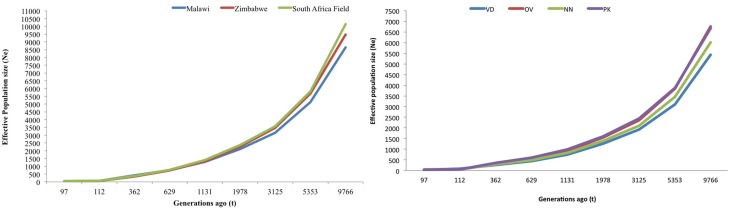
**(A)** Trends in effective population size of village chicken flocks. **(B)** Trends in effective population size of conservation flocks. VD, Venda conservation flocks; NN, Naked Neck conservation flocks; OV, Ovambo conservation flocks; PK, Potchefstroom Koekoek conservation flock.

Figures 5A,B are plots of the estimated effective population size (N_e_) at *t* generations ago for the village and conservation flocks, respectively. The adjusted LD based estimates of N_e_ indicated low effective population size of 49–57 in the village chickens and of 31–50 in the conservation flocks 97 generations ago. The graphs also illustrate a steady decrease in effective population size from over 8500 to below 60 within 9000 generations for the village chickens (Figure 5A) and from 6000 to below 50, during the same time frame, for the conservation flocks (Figure 5B).

## Discussion

Village chicken populations in sub-Saharan Africa have not been well studied to estimate genetic and demographic parameters that are shaping their genetic structure. Previous studies have suggested that village chickens are a valuable genetic reservoir, particularly for smallholder resource-limited farmers, due to their ability to thrive in diverse geographical environments characterized by extreme climatic conditions (Hall and Bradley, 1995). Random mating and absence of pedigree data make it difficult to estimate the effective population size and other key population genetic parameters in these populations. In addition, absence of record keeping and organization hinder prospects of conducting genetic improvement programs required to improve phenotypes. Previous genetic diversity studies of village chickens utilized microsatellite markers (Muchadeyi et al., 2007; Mtileni et al., 2011b) as well as mitochondrial DNA (mtDNA) (Mobegi et al., 2006; Adebambo and Consortium, 2009; rnstad, Mobegi, Nomura, Hanada and Amano, Mwacharo et al., 2011; Wani et al., 2014). Microsatellite markers are very informative but too sparse over the genome to provide accurate estimates of population genetic parameters. MtDNA sequences are informative for investigating species domestication and migration but they lack genome-wide coverage (Godinho et al., 2008). High density SNP chips have been successfully used in recent studies to characterize LD (Megens et al., 2009; Qanbari et al., 2010) and Mendelian traits and screen for other genetic variants in commercial layers (Qanbari et al., 2010) and in traditional chicken populations raised under production systems similar to those of our village chickens (Wragg et al., 2012). However, there is no information on the utility of this panel of markers for village chickens from the Southern African region. This study therefore used genome-wide SNP data to estimate population structure and diversity, linkage disequilibrium and population demographic history of extensively raised chicken populations of Southern Africa.

Only 51% of the SNPs on the iselect chicken SNP60K panel were used for the estimation of population structure and diversity. The remaining SNPs were excluded because they had MAF below the set threshold (MAF ≤0.02) or were in linkage disequilibrium. Conversely, over 80% of the SNPs of the panel were used for LD analysis. The number of monomorphic markers observed in this study was about 5 fold lower than those reported in Qanbari et al. (2010) in commercial layers, and 2–3 fold higher than that reported by Wragg et al. (2012) in traditional village chickens from Ethiopia, Kenya, and Chile. Even if variations in the number of monomorphic markers can be partially explained by the different number of animals sampled, it seems clear that village chickens hold a higher diversity than commercial populations and that the 60K SNP chip has some utility in genomics studies of non-descript chicken populations in spite of the ascertainment bias embedded in its design.

Population structure analysis grouped the conservation flocks into four distinct clusters that were different from the village chickens sampled from the communal farming areas of South Africa, Zimbabwe, and Malawi (Figures 2, 3). This study and that of Mtileni et al. (2011b), which was based on microsatellite markers, showed that the conservation flocks have diverged from their founder village chicken populations. The levels of population divergence showed the Venda and Naked Neck were more distant to the South African village chickens than the Potchefstroom Koekoek and Ovambo chickens. Variations in levels of population divergence could have originated from different founder effects and reduced population sizes in these conservation flocks. The clustering of populations from both the PCA and ADMIXTURE indicated low levels of within population diversity of the Venda and Naked Neck conservation flocks and higher divergences of these populations from the Ovambo, Potchefstroom Koekoek and village flocks. This observation was also supported by the relatively higher heterozygosity deficiency and inbreeding coefficients of the Venda and Naked Neck conservation flocks (Table 1).

Conservation chickens were sampled from closed populations, kept at the Agricultural Research Council in South Africa, ranging from 100 to 150 chickens/flock (van Marle-Köster et al., 2008; Mtileni et al., 2011a) that have a narrow genetic base. The flocks were established from chickens sampled from villages in South Africa. The Venda chicken flocks were established from a few individuals based on rare plumage color variants amongst other diverse phenotypes in the Limpopo province of South Africa (van Marle-Köster et al., 2008). The Naked Neck, as their name implies, were similarly sub-sampled from a heterogenous pool of village chickens in the Eastern Cape provinces of South Africa for this phenotype caused by a single gene that is dominantly expressed, and is considered to be one of only a few distinct phenotypes observed in most village chickens in South Africa and other developing countries. On the other hand, the Ovambo chickens were established from a representative sample of village chickens found in the Ovambo regions bordering South Africa and Namibia (van Marle-Köster et al., 2008). The actual numbers of individual chickens used to establish the Naked-Neck, Venda, and Ovambo populations are not known. The Potchefstroom Koekoek was established by crossing a number of lines of White Leghorn females and Black Australorp males. The Barred Plymouth Rock was later introduced to the breeding program (Viljoen, 1986) giving this flock a relatively a broader founder population compared to the other flocks.

Whilst it is evident that the conservation flocks diverged from the village chicken populations they were founded from, results from ADMIXTURE indicated that the Potchefstroom Koekoek and Naked Neck have retained single and unique ancestral genomic components from the founder flocks (Figure 3) and could be used to conserve part of the genetic diversity found in the village chickens. In contrast, the Venda conservation flock has evolved into a population with a completely different genomic composition to that of the village chickens. Of the four flocks, the Ovambo chickens appear to have maintained much of the village chicken genetic diversity and could therefore be a good and more representative conservation flock.

The village chicken samples were obtained from multiple agro-ecological zones within a country except for the chickens of Malawi that were obtained from a single agro-ecological zone. The clustering of the village chickens followed a geographical gradient in which the South African chickens were least related to the Malawian chickens. Higher levels of divergence between village chickens from Malawi and South Africa could be explained by geographical distance from each other, lack of gene flow between the two countries and isolated evolution occurring in these populations. Village chickens of South Africa and Zimbabwe had more within population diversity, as indicated by their wide spread clusters, than the conservation flocks. This could be due to a combination of founder effects in the conservation flocks as well as gene flow between the two countries.

Linkage disequilibrium was calculated using 28 of the 38 chicken autosomal chromosomes that were represented on the Illumina iSelect SNP60K bead chip. The 10 autosomes not used for this analysis are micro chromosomes that were not included in the design of the 60K bead chip as they were not yet covered by the genome build *Gallus gallus* v2.1 (Groenen et al., 2011). SNPs on linkage groups and sex chromosomes as well as those of unknown marker positions were excluded from the analysis. Most SNPs were pruned due to monomorphism and minor allele frequency. A threshold of MAF ≤0.05 was used prior to LD analysis in this and other studies (Qanbari et al., 2010; Wragg et al., 2012) which, according to Corbin et al. (2010), can increase accuracy on LD measures when sample size is large. It was observed by Corbin et al. (2010) and Corbin et al. (2012) that pruning MAF of more than 0.1 can lead to ascertainment bias on the measures of effective population size particularly in small to moderate sample sizes.

The overall LD values between populations showed significant differences between populations with higher LD observed in the conservation flocks and low LD in the village chicken populations kept by smallholder farmers. Variation in LD between the conservation flocks and village chicken populations could be an indication of different population histories and the influences of different evolutionary mechanisms in terms of bottleneck effect, genetic drift, selection and mutations in different population categories. The least diverse (Table 1) and highly divergent (Figures 2, 3) Venda flock was also observed to have high LD compared to the other populations which implies low effective population size and diversity of this population as suggested by the population structure based methods. LD was consistently high on chromosome 16 and was low on chromosome 25 for both village and conservation flocks. The chromosomal difference in LD supports observations by Andreescu et al. (2007), Megens et al. (2009), and Qanbari et al. (2010). However, studies by Andreescu et al. (2007) and Megens et al. (2009) focused on selected genomic regions and selected chromosomes. Findings from the current study and studies by Megens et al. (2009) indicate that evolutionary forces affecting LD act differently on different chromosomes within populations. Natural selection could be a major factor in village chicken populations that are raised under extensive systems characterized by low production levels and minimal human selection pressures (Mtileni et al., 2010).

The current study also indicated a significant LD decay with increased marker intervals, which generally is a function of increased recombination events with increased genetic distance (Megens et al., 2009). The high GC content and high density of genes on micro-chromosomes compared to macro-chromosomes is also associated with high recombination events, which results in lower LD (Megens et al., 2009) and the current results agreed with the expected trends.

Over and above the expected trends in LD decay with increased marker distances, LD was moderately high and remained well above 0.2 at marker distances of up to 500 kb when using genomewide SNP data and upto 1000 kb for macro-chromosomes 1–5 in the conservation flocks (Figures 4A,B). On the other hand, LD decayed to relatively lower values below 0.1 in the village chicken populations. The relatively high average LD that starts at very short marker distance of 10 kb and is persistent over long distances could be a reflection of low effective population size in the conservation vs. village chicken populations which will be in agreement with results on population structure (Figures 2, 3) and other population diversity analysis (Table 1).

Analysis of trends in effective population size from LD values suggested low effective population sizes particularly in the conservation flocks. Results showed a decrease in genetic variation over time in both conservation and village chicken flocks which could be due to poor management, inbreeding as a result of population sub-structuring within villages or population bottlenecks that could have been experienced during the development of these populations (Figures 5A,B). The overlapping generations in smallholder farming systems promote mating of closely related chickens thereby increasing inbreeding levels (which were high in conservation flocks, Table 1). On the other hand, although village farmers are known to keep small flocks ranging from 1 to 20 chicken per household significant levels of cock sharing is expected within villages which could actually result in higher effective population sizes. Results from microsatellite analyses (Muchadeyi et al., 2007; Mtileni et al., 2011b) have suggested a high level of population diversity within village chicken populations.

Overall, the study demonstrated the utility of the Illumina chicken iselect SNP 60K panel in extensively raised and conservation flocks with limitations due to high proportion of monomorphic and less polymorphic SNPs. Only a subset of independent SNPs could be used for population structure analysis. The study observed population divergence resulting in clear population boundaries between the conservation and the village flocks. High levels of population diversity were observed in the village chickens as well as the Ovambo conservation flock. A relatively high LD that persisted over longer SNP intervals was observed in the South African conservation flocks and not the village chicken populations. This LD pattern seems to be consistent with low effective population sizes and loss of diversity in conservation populations which could be an effect of small size of the founder populations and them being raised as closed populations prone to the effects of inbreeding and genetic drift.

## Author contributions

Khulekani S. Khanyile carried out the laboratory analyses, statistical analyses, and interpretation of the data and drafted the manuscript. Farai C. Muchadeyi and Edgar F. Dzomba assisted with the acquisition of funding, designing and execution of the experiment and revised the manuscript critically for important intellectual content.

## Conflict of interest statement

The authors declare that the research was conducted in the absence of any commercial or financial relationships that could be construed as a potential conflict of interest.
