# Supplementary figures and images for: Population genetic structure, linkage disequilibrium and effective population size of conserved and extensively raised village chicken populations of Southern Africa
Source: Front Genet. 2015 Feb 3;6:13. doi: 10.3389/fgene.2015.00013 (PMC4315093; doi:10.3389/fgene.2015.00013)

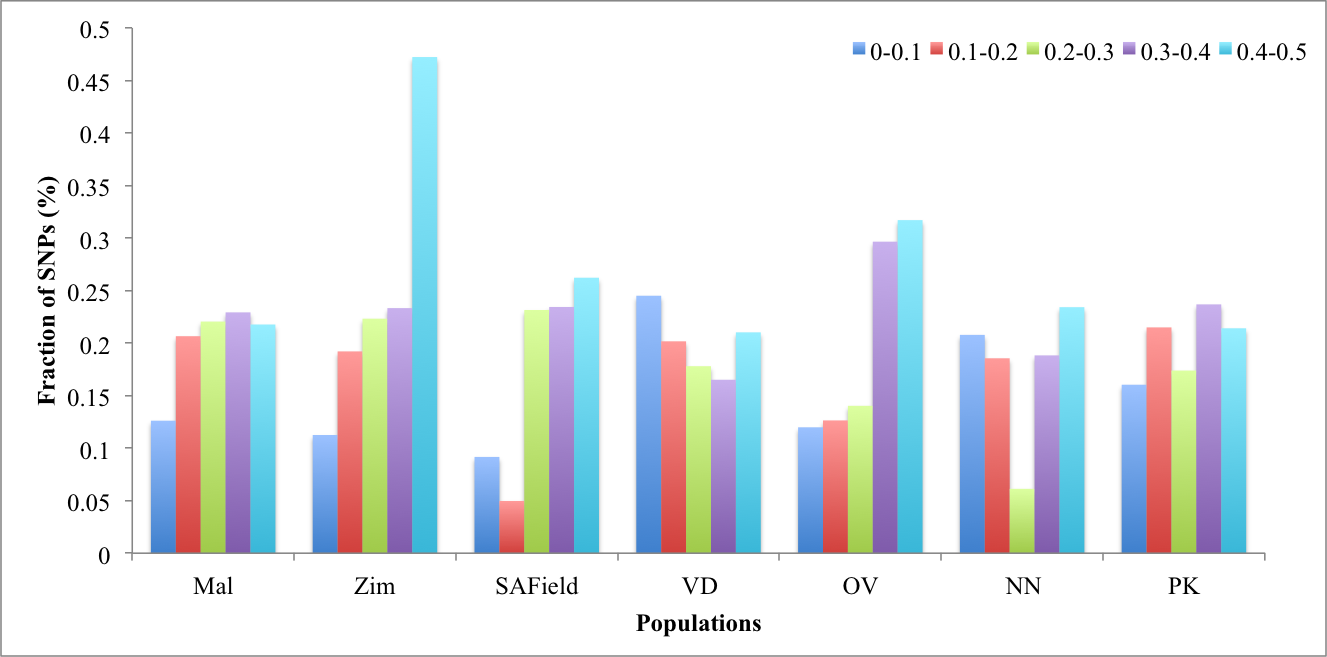

Supplement: Supplementary file 1 [file Image1.TIFF]

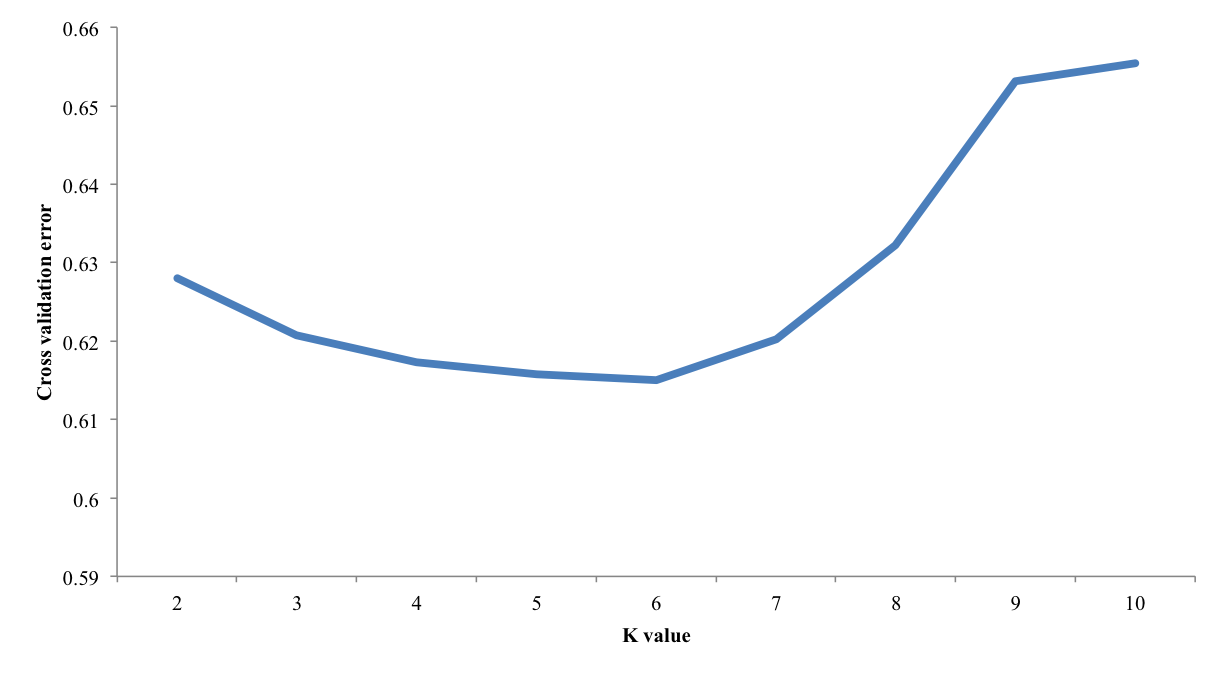

Supplement: Supplementary file 2 [file Image2.TIFF]
