# Supplementary material for: Population genetic structure, linkage disequilibrium and effective population size of conserved and extensively raised village chicken populations of Southern Africa
Source: Front Genet. 2015 Feb 3;6:13. doi: 10.3389/fgene.2015.00013 (PMC4315093; doi:10.3389/fgene.2015.00013)
Supplement: Supplementary file 3 [file Image3.PDF]

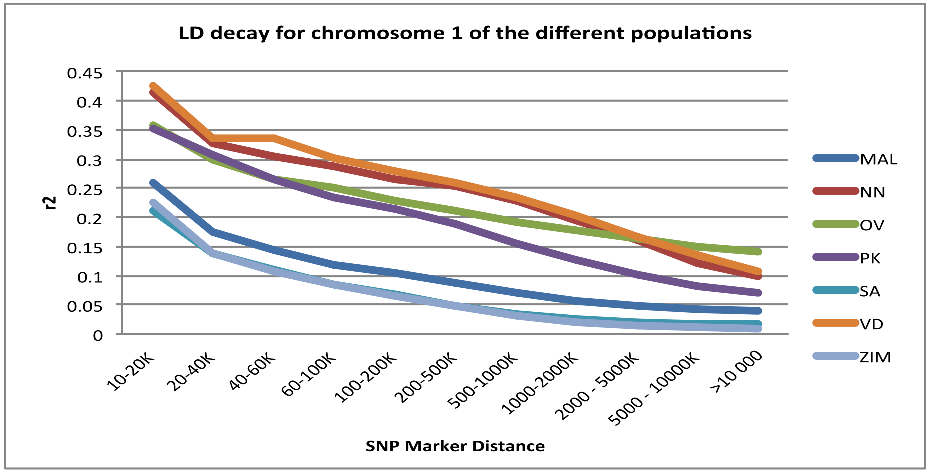
LD decay for chromosome 1 of the different populations
r2
0.45
0.4
0.35
0.3
0.25
0.2
0.15
0.1
0.05
0
10-20K
20-40K
40-60K
60-100K
100-200K
200-500K
500-1000K
1000-2000K
2000 - 5000K
5000 - 10000K
>10 000
SNP Marker Distance
MAL
NN
OV
PK
SA
VD
ZIM

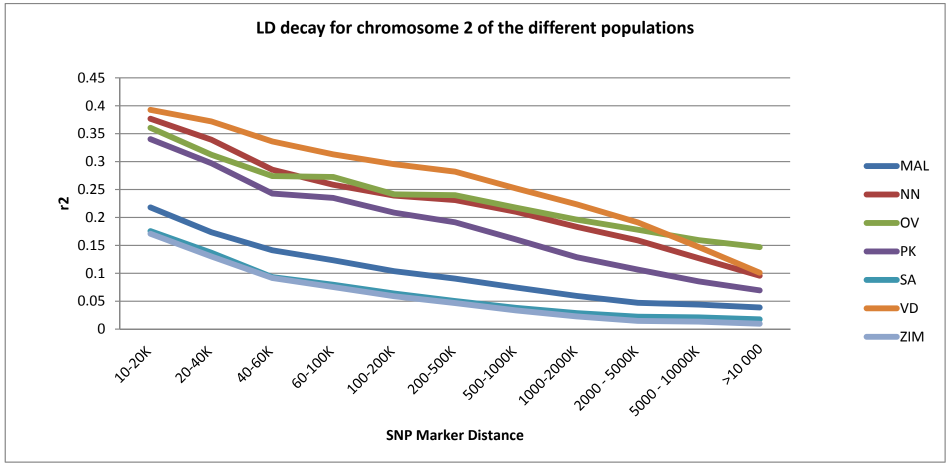
LD decay for chromosome 2 of the different populations
r2
0.45
0.4
0.35
0.3
0.25
0.2
0.15
0.1
0.05
0
10-20K
20-40K
40-60K
60-100K
100-200K
200-500K
500-1000K
1000-2000K
2000 - 5000K
5000 - 10000K
>10 000
SNP Marker Distance
MAL
NN
OV
PK
SA
VD
ZIM

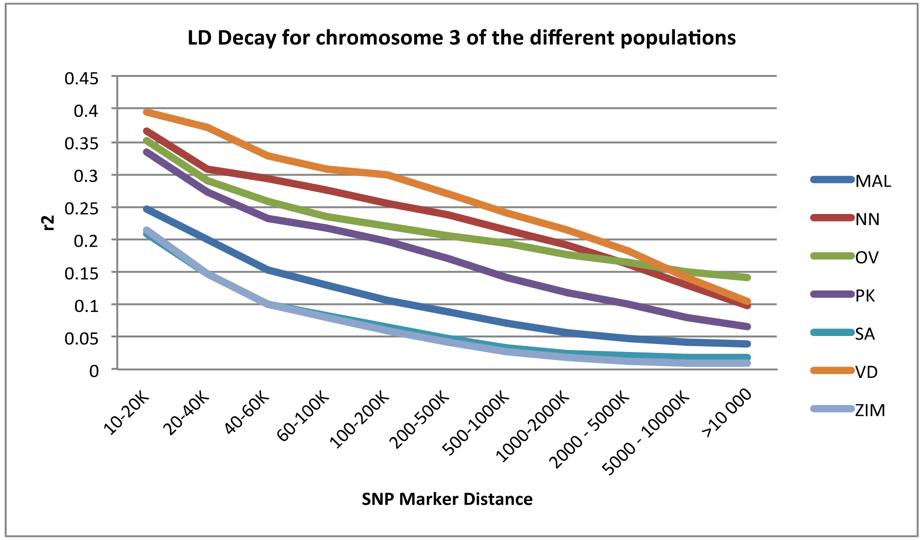
LD Decay for chromosome 3 of the different populations
r2
0.45
0.4
0.35
0.3
0.25
0.2
0.15
0.1
0.05
0
10-20K
20-40K
40-60K
60-100K
100-200K
200-500K
500-1000K
1000-2000K
2000 - 5000K
5000 - 10000K
>10 000
SNP Marker Distance
MAL
NN
OV
PK
SA
VD
ZIM

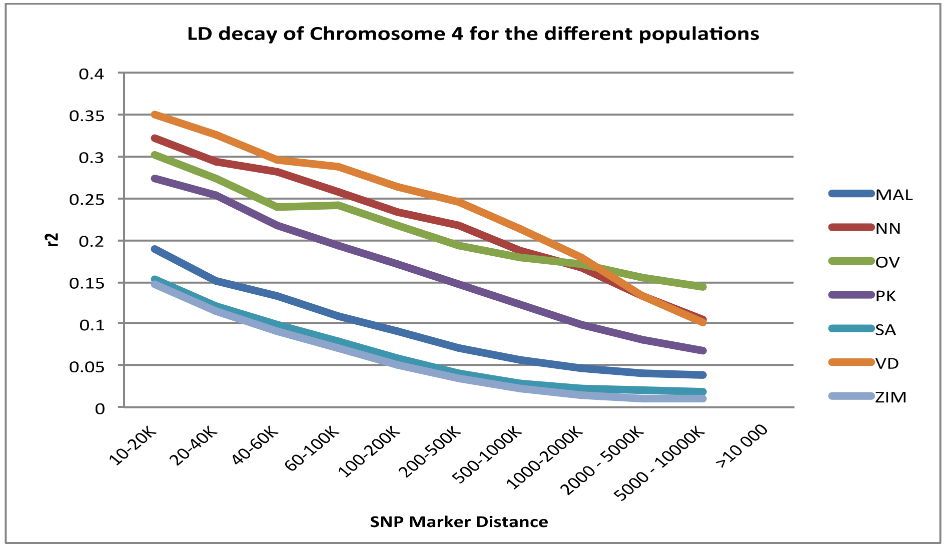
LD decay of Chromosome 4 for the different populations
r2
0.4
0.35
0.3
0.25
0.2
0.15
0.1
0.05
0
10-20K
20-40K
40-60K
60-100K
100-200K
200-500K
500-1000K
1000-2000K
2000 - 5000K
5000 - 10000K
>10 000
SNP Marker Distance
MAL
NN
OV
PK
SA
VD
ZIM

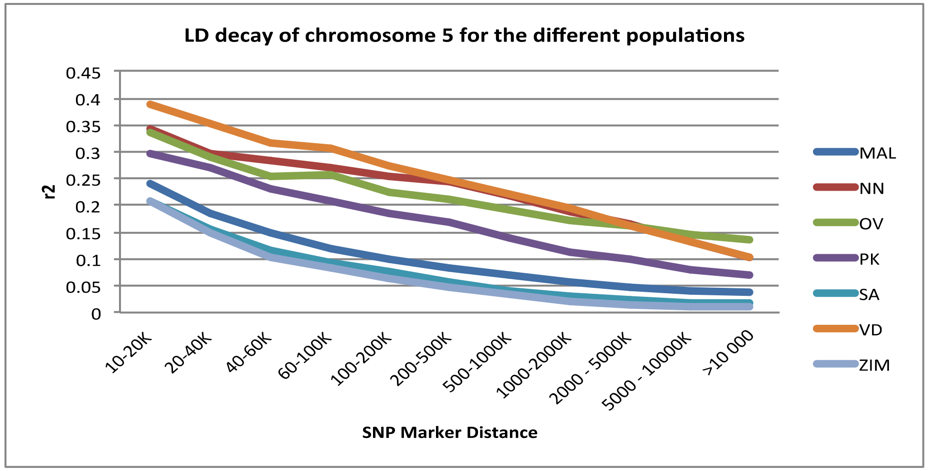

LD decay of chromosome 5 for the different populations
r2
0.45
0.4
0.35
0.3
0.25
0.2
0.15
0.1
0.05
0
10-20K
20-40K
40-60K
60-100K
100-200K
200-500K
500-1000K
1000-2000K
2000 - 5000K
5000 - 10000K
>10 000
SNP Marker Distance
MAL
NN
OV
PK
SA
VD
ZIM

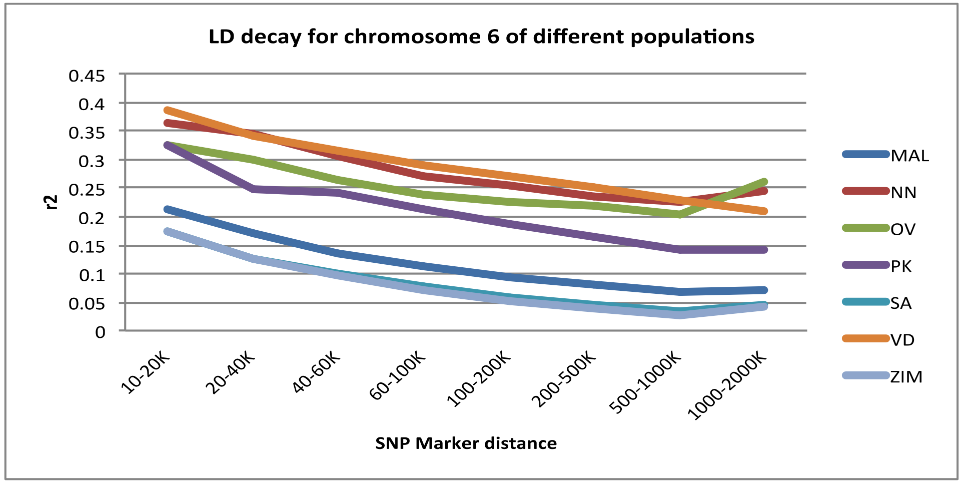

LD decay for chromosome 6 of different populations
r2
0.45
0.4
0.35
0.3
0.25
0.2
0.15
0.1
0.05
0
10-20K
20-40K
40-60K
60-100K
100-200K
200-500K
500-1000K
1000-2000K
SNP Marker distance
MAL
NN
OV
PK
SA
VD
ZIM

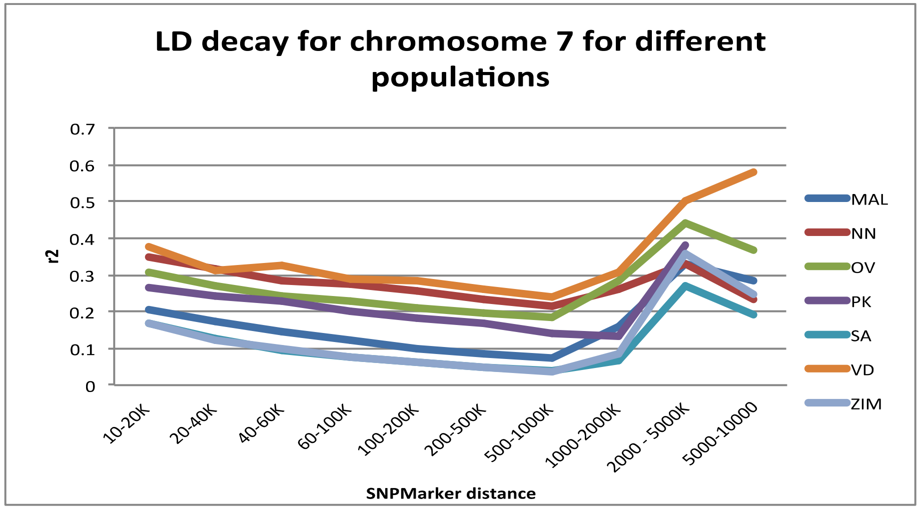

LD decay for chromosome 7 for different populations
r2
0.7
0.6
0.5
0.4
0.3
0.2
0.1
0
10-20K
20-40K
40-60K
60-100K
100-200K
200-500K
500-1000K
1000-2000K
2000 - 5000K
5000-10000
SNPMarker distance
MAL
NN
OV
PK
SA
VD
ZIM

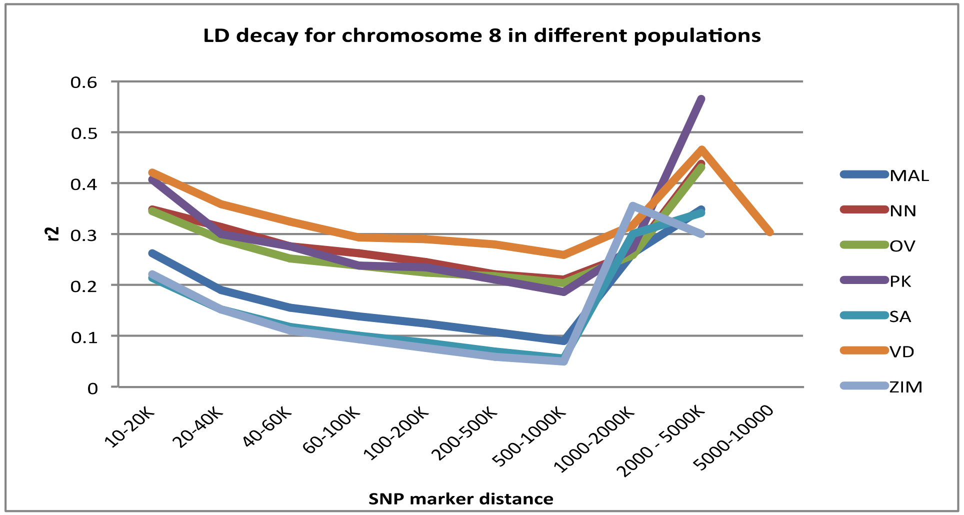

LD decay for chromosome 8 in different populations
r2
0.6
0.5
0.4
0.3
0.2
0.1
0
10-20K
20-40K
40-60K
60-100K
100-200K
200-500K
500-1000K
1000-2000K
2000 - 5000K
5000-10000
SNP marker distance
MAL
NN
OV
PK
SA
VD
ZIM

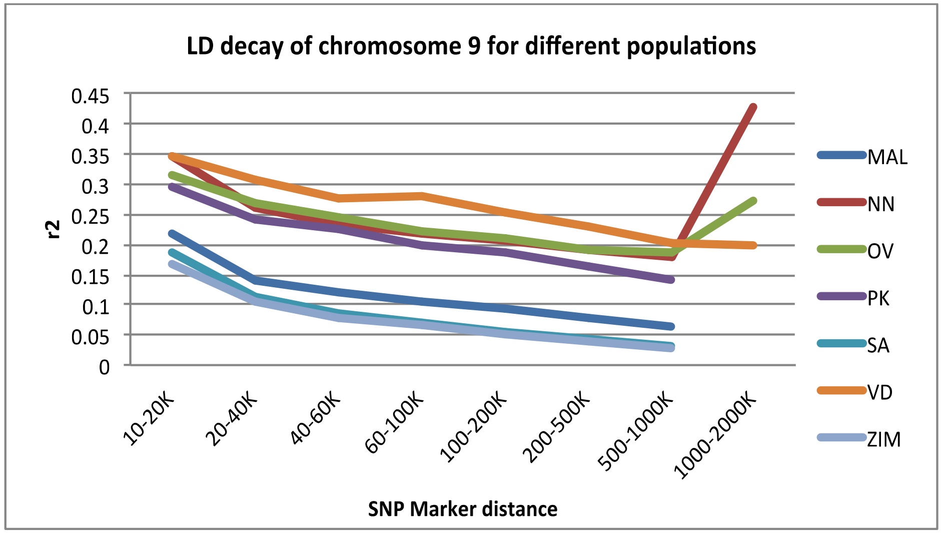
LD decay of chromosome 9 for different populations
0.45
0.4
0.35
0.3
0.25
0.2
0.15
0.1
0.05
0
r2
10-20K
20-40K
40-60K
60-100K
100-200K
200-500K
500-1000K
1000-2000K
SNP Marker distance
MAL
NN
OV
PK
SA
VD
ZIM

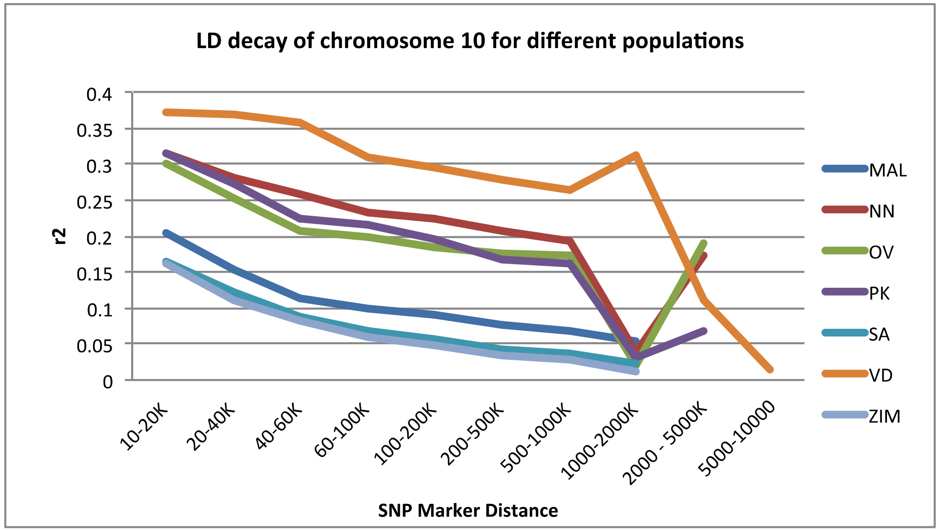
LD decay of chromosome 10 for different populations
0.4
0.35
0.3
0.25
0.2
0.15
0.1
0.05
0
r2
10-20K
20-40K
40-60K
60-100K
100-200K
200-500K
500-1000K
1000-2000K
2000 - 5000K
5000-10000
SNP Marker Distance
MAL
NN
OV
PK
SA
VD
ZIM

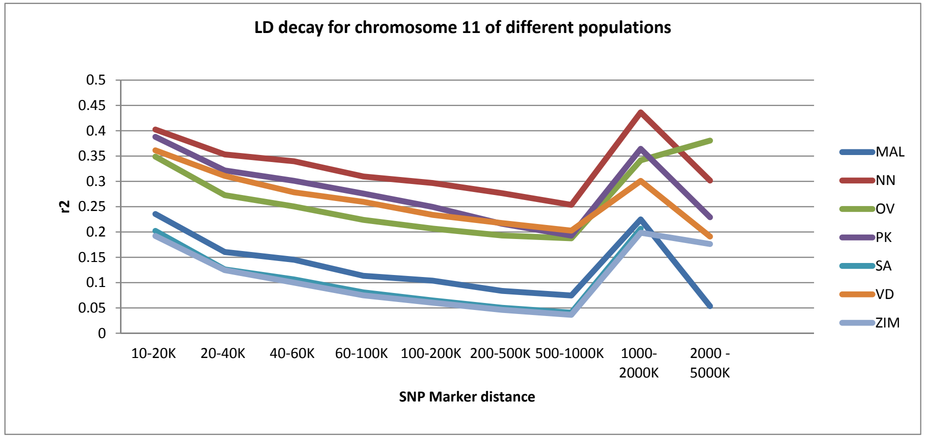
LD decay for chromosome 11 of different populations
0.5
0.45
0.4
0.35
0.3
0.25
0.2
0.15
0.1
0.05
0
r2
10-20K
20-40K
40-60K
60-100K
100-200K
200-500K
500-1000K
1000-2000K
2000 - 5000K
SNP Marker distance
MAL
NN
OV
PK
SA
VD
ZIM

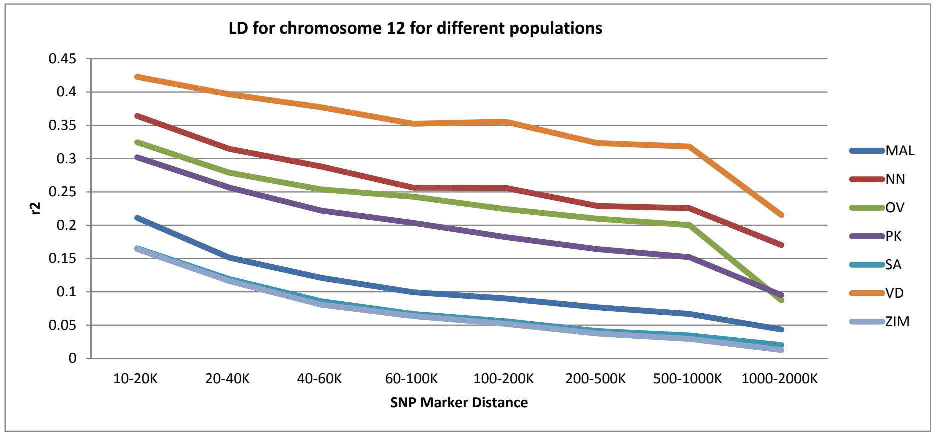
LD for chromosome 12 for different populations
0.45
0.4
0.35
0.3
0.25
0.2
0.15
0.1
0.05
0
r2
10-20K
20-40K
40-60K
60-100K
100-200K
200-500K
500-1000K
1000-2000K
SNP Marker Distance
MAL
NN
OV
PK
SA
VD
ZIM

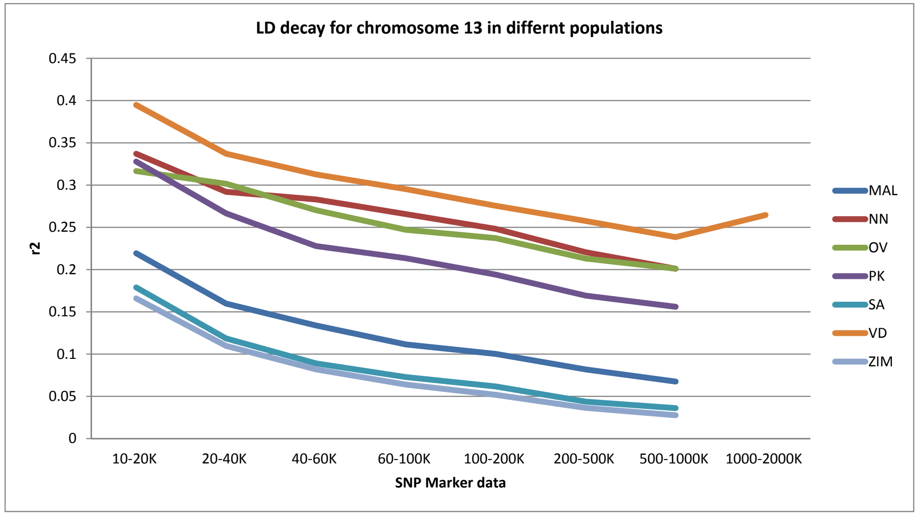
LD decay for chromosome 13 in differnt populations
r2
0.45
0.4
0.35
0.3
0.25
0.2
0.15
0.1
0.05
0
10-20K
20-40K
40-60K
60-100K
100-200K
200-500K
500-1000K
1000-2000K
SNP Marker data
MAL
NN
OV
PK
SA
VD
ZIM

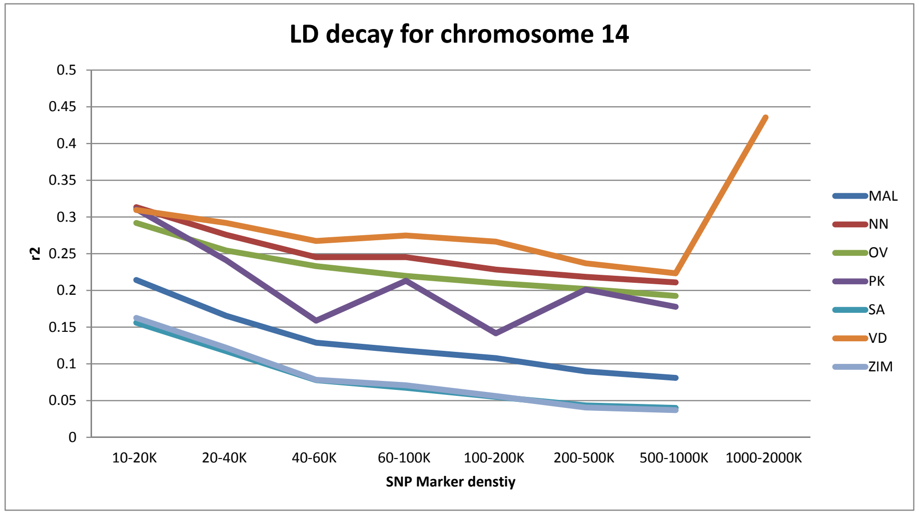
LD decay for chromosome 14
r2
0.5
0.45
0.4
0.35
0.3
0.25
0.2
0.15
0.1
0.05
0
10-20K
20-40K
40-60K
60-100K
100-200K
200-500K
500-1000K
1000-2000K
SNP Marker denstiy
MAL
NN
OV
PK
SA
VD
ZIM

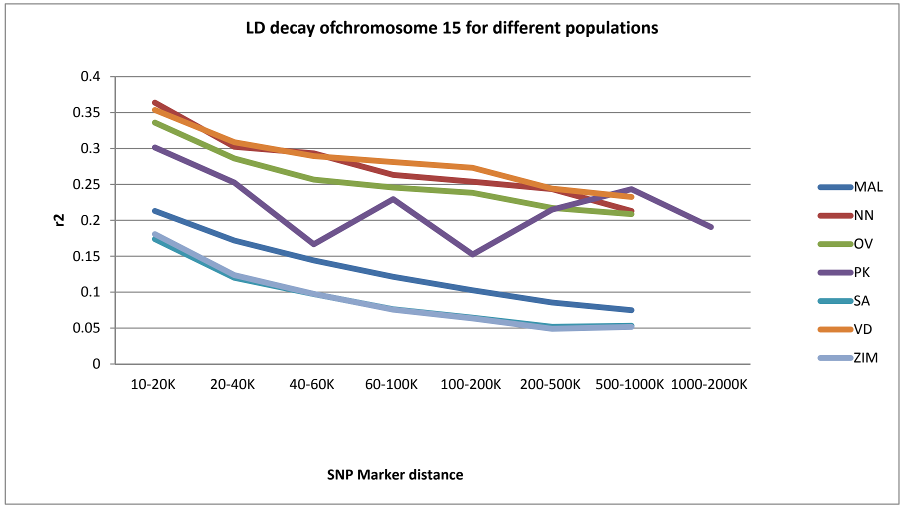
LD decay ofchromosome 15 for different populations
r2
0.4
0.35
0.3
0.25
0.2
0.15
0.1
0.05
0
10-20K
20-40K
40-60K
60-100K
100-200K
200-500K
500-1000K
1000-2000K
SNP Marker distance
MAL
NN
OV
PK
SA
VD
ZIM

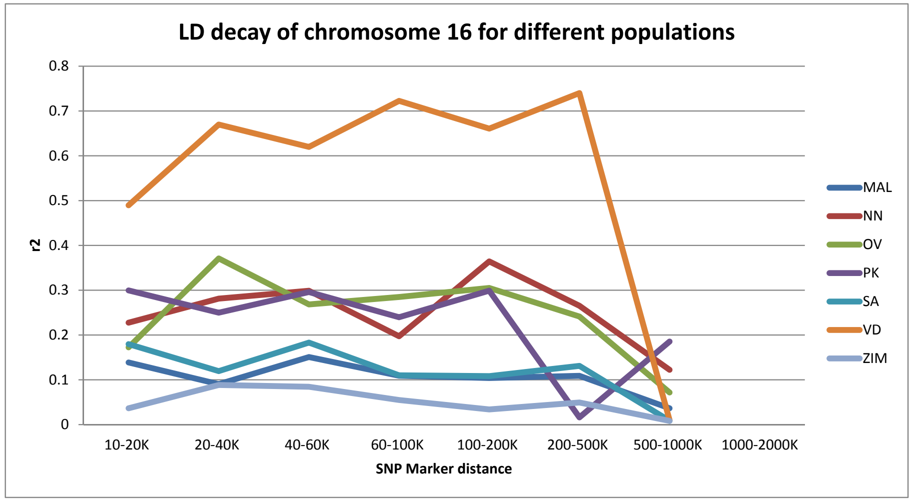
LD decay of chromosome 16 for different populations
r2
0.8
0.7
0.6
0.5
0.4
0.3
0.2
0.1
0
10-20K
20-40K
40-60K
60-100K
100-200K
200-500K
500-1000K
1000-2000K
SNP Marker distance
MAL
NN
OV
PK
SA
VD
ZIM

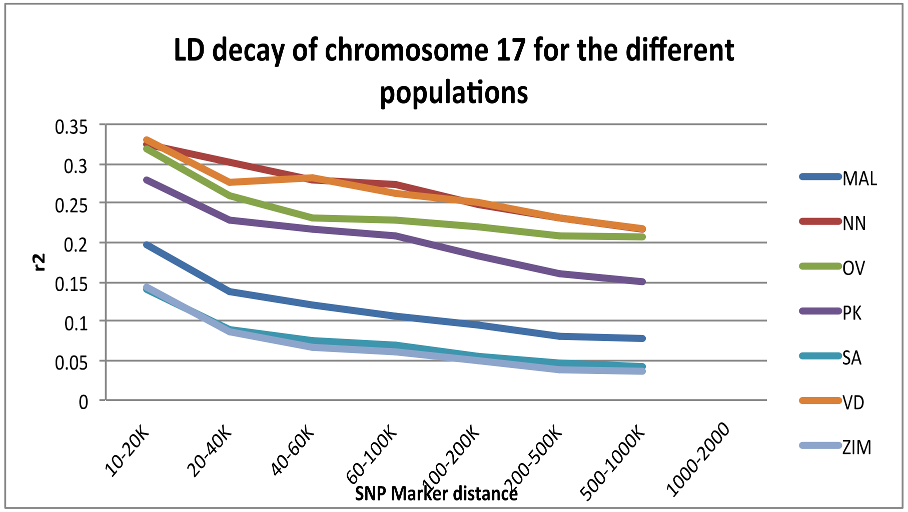
LD decay of chromosome 17 for the different populations
r2
0.35
0.3
0.25
0.2
0.15
0.1
0.05
0
10-20K
20-40K
40-60K
60-100K
100-200K
200-500K
500-1000K
1000-2000
SNP Marker distance
MAL
NN
OV
PK
SA
VD
ZIM

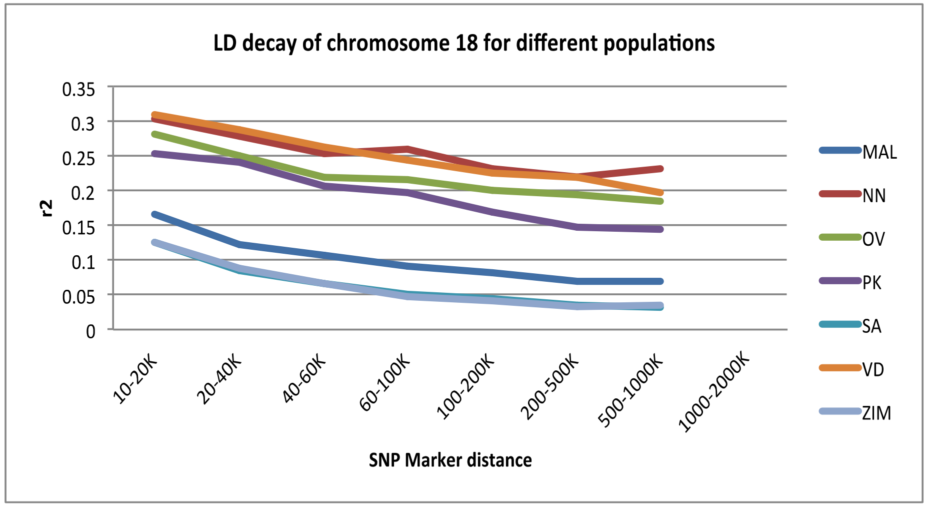
LD decay of chromosome 18 for different populations
r2
0.35
0.3
0.25
0.2
0.15
0.1
0.05
0
10-20K
20-40K
40-60K
60-100K
100-200K
200-500K
500-1000K
1000-2000K
SNP Marker distance
MAL
NN
OV
PK
SA
VD
ZIM

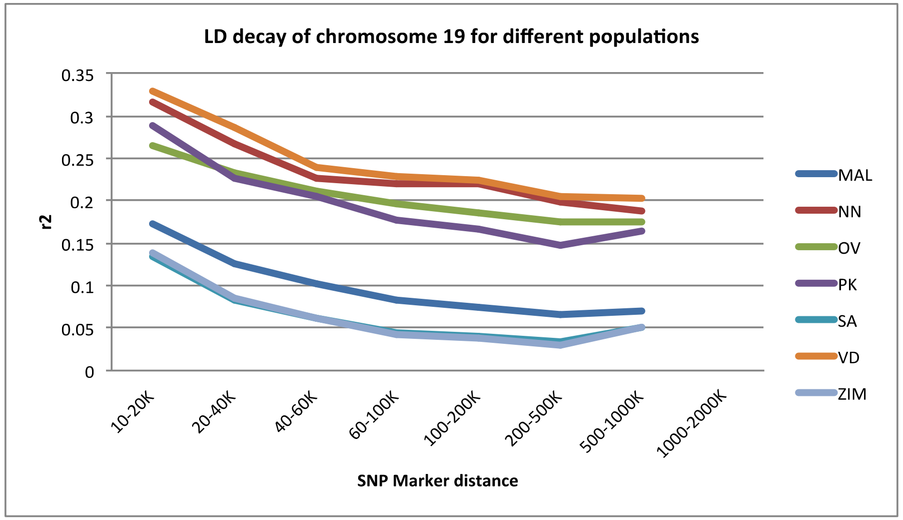
LD decay of chromosome 19 for different populations
r2
0.35
0.3
0.25
0.2
0.15
0.1
0.05
0
10-20K
20-40K
40-60K
60-100K
100-200K
200-500K
500-1000K
1000-2000K
SNP Marker distance
MAL
NN
OV
PK
SA
VD
ZIM

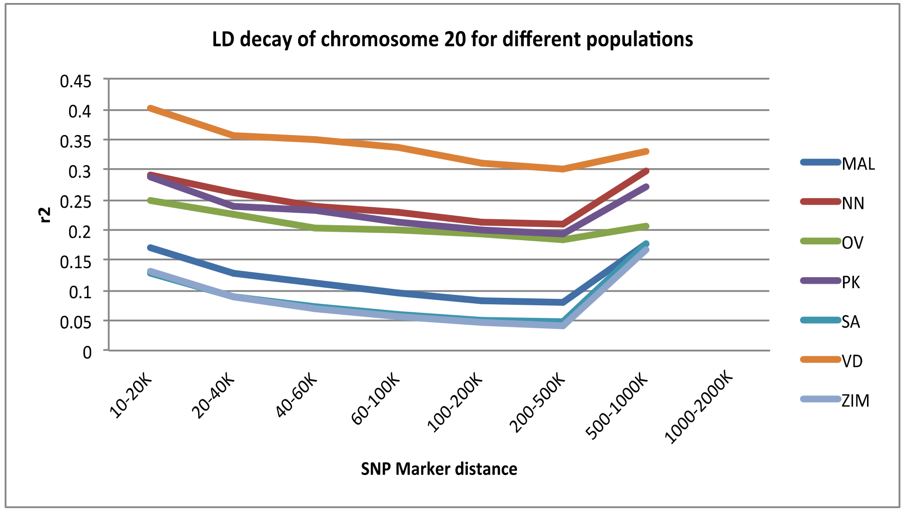
LD decay of chromosome 20 for different populations
r2
0.45
0.4
0.35
0.3
0.25
0.2
0.15
0.1
0.05
0
10-20K
20-40K
40-60K
60-100K
100-200K
200-500K
500-1000K
1000-2000K
SNP Marker distance
MAL
NN
OV
PK
SA
VD
ZIM

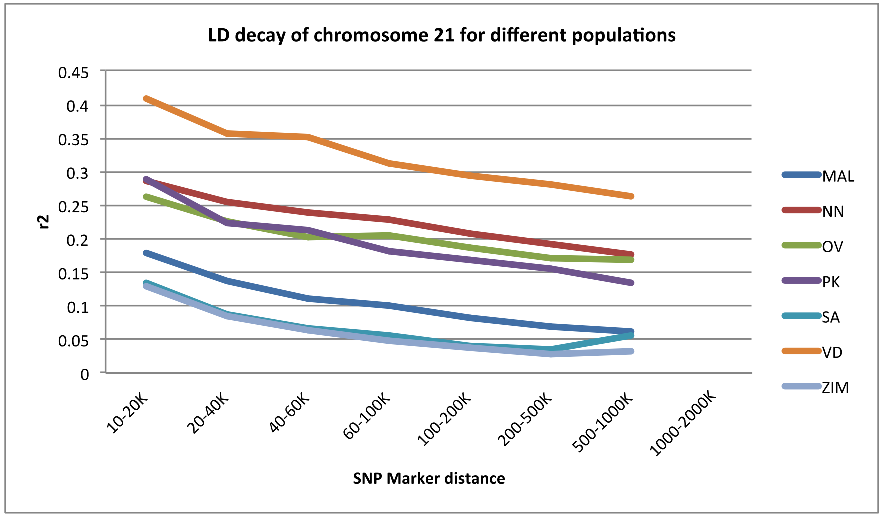
LD decay of chromosome 21 for different populations
r2
0.45
0.4
0.35
0.3
0.25
0.2
0.15
0.1
0.05
0
10-20K
20-40K
40-60K
60-100K
100-200K
200-500K
500-1000K
1000-2000K
SNP Marker distance
MAL
NN
OV
PK
SA
VD
ZIM

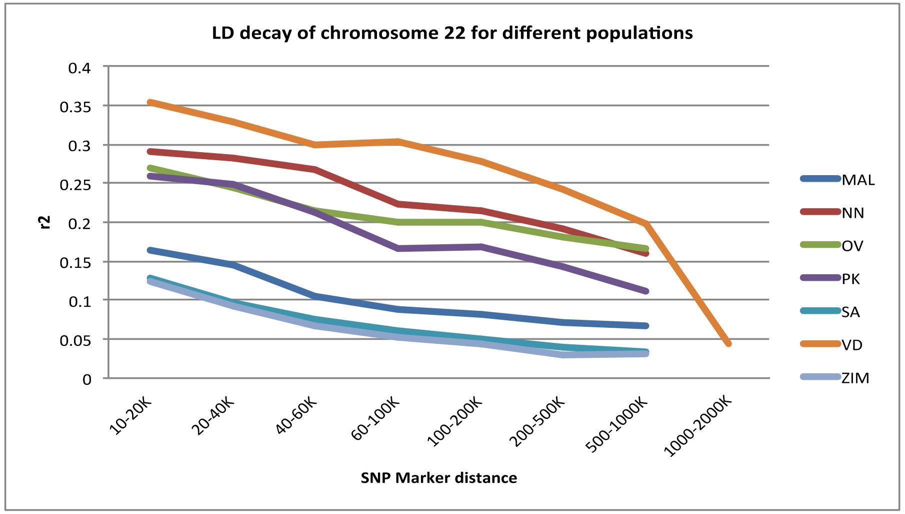
LD decay of chromosome 22 for different populations
r2
0.4
0.35
0.3
0.25
0.2
0.15
0.1
0.05
0
10-20K
20-40K
40-60K
60-100K
100-200K
200-500K
500-1000K
1000-2000K
SNP Marker distance
MAL
NN
OV
PK
SA
VD
ZIM

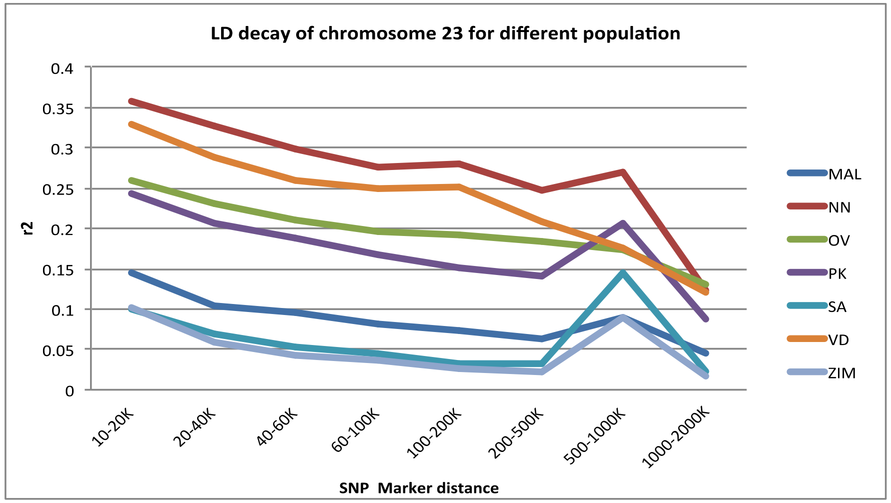
LD decay of chromosome 23 for different population
r2
0.4
0.35
0.3
0.25
0.2
0.15
0.1
0.05
0
10-20K
20-40K
40-60K
60-100K
100-200K
200-500K
500-1000K
1000-2000K
SNP Marker distance
MAL
NN
OV
PK
SA
VD
ZIM

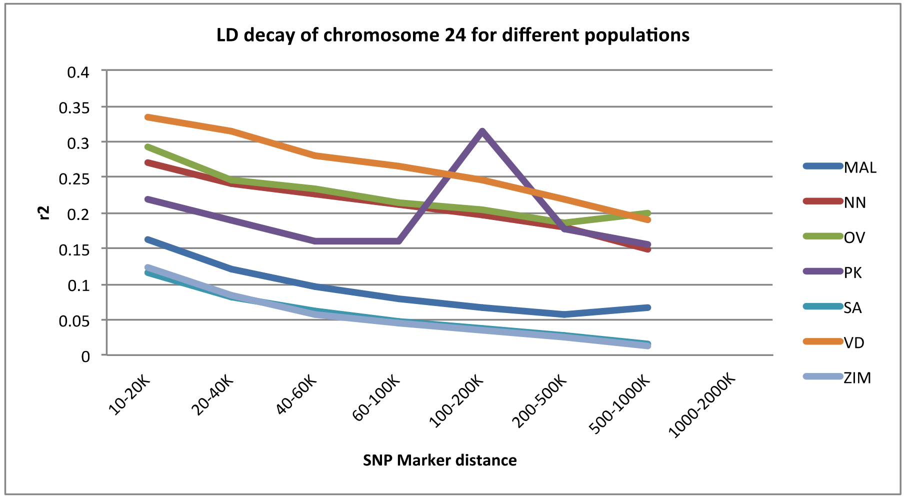
LD decay of chromosome 24 for different populations
r2
0.4
0.35
0.3
0.25
0.2
0.15
0.1
0.05
0
10-20K
20-40K
40-60K
60-100K
100-200K
200-500K
500-1000K
1000-2000K
SNP Marker distance
MAL
NN
OV
PK
SA
VD
ZIM

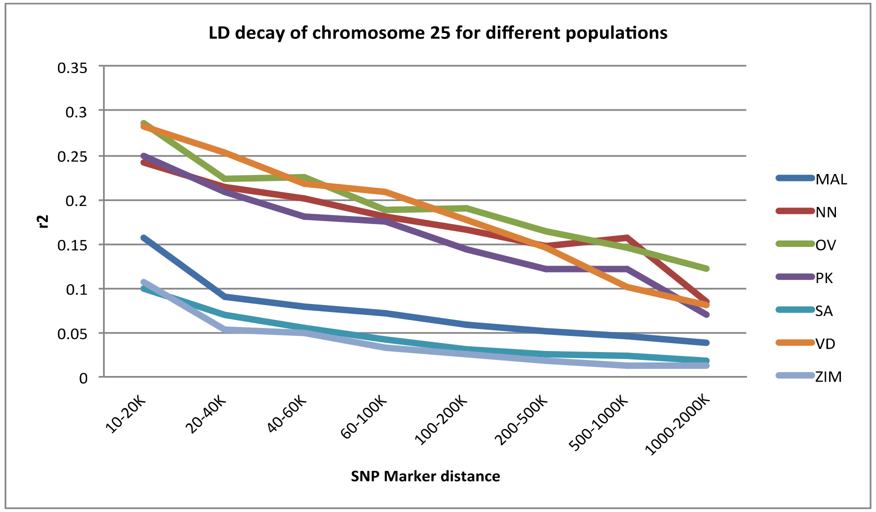

LD decay of chromosome 25 for different populations
0.35
0.3
0.25
0.2
0.15
0.1
0.05
0
r2
10-20K
20-40K
40-60K
60-100K
100-200K
200-500K
500-1000K
1000-2000K
SNP Marker distance
MAL
NN
OV
PK
SA
VD
ZIM

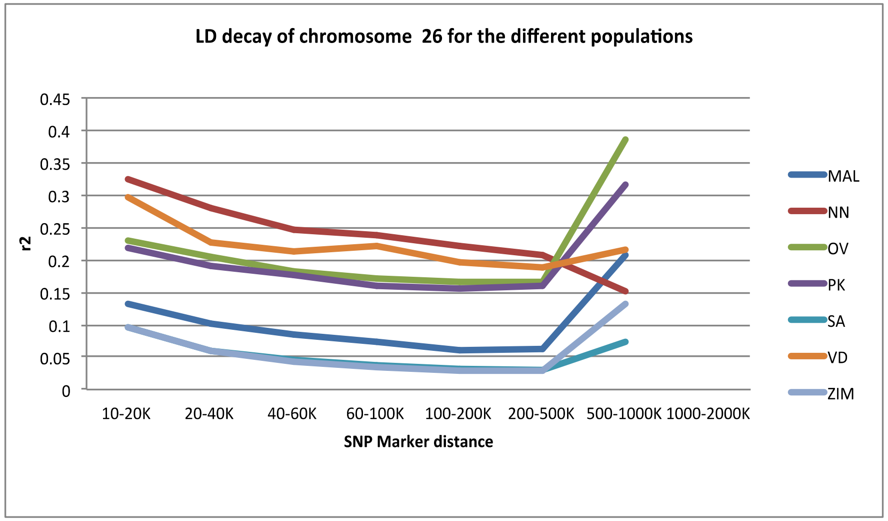

LD decay of chromosome 26 for the different populations
0.45
0.4
0.35
0.3
0.25
0.2
0.15
0.1
0.05
0
r2
10-20K
20-40K
40-60K
60-100K
100-200K
200-500K
500-1000K
1000-2000K
SNP Marker distance
MAL
NN
OV
PK
SA
VD
ZIM

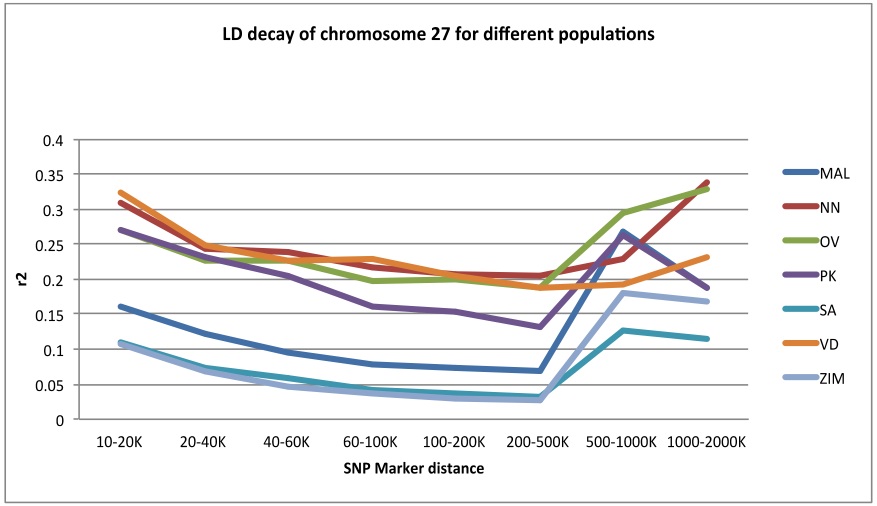

LD decay of chromosome 27 for different populations
0.4
0.35
0.3
0.25
0.2
0.15
0.1
0.05
0
r2
10-20K
20-40K
40-60K
60-100K
100-200K
200-500K
500-1000K
1000-2000K
SNP Marker distance
MAL
NN
OV
PK
SA
VD
ZIM

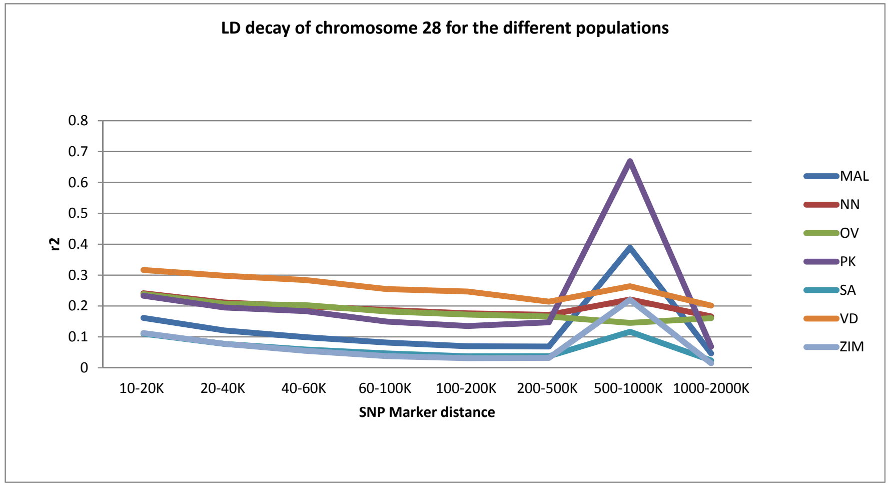

LD decay of chromosome 28 for the different populations
0.8
0.7
0.6
0.5
0.4
0.3
0.2
0.1
0
r2
10-20K
20-40K
40-60K
60-100K
100-200K
200-500K
500-1000K
1000-2000K
SNP Marker distance
MAL
NN
OV
PK
SA
VD
ZIM
