# Supplementary material for: Population genetic structure, linkage disequilibrium and effective population size of conserved and extensively raised village chicken populations of Southern Africa
Source: Front Genet. 2015 Feb 3;6:13. doi: 10.3389/fgene.2015.00013 (PMC4315093; doi:10.3389/fgene.2015.00013)
Supplement: Supplementary file 4 [file Table1.DOCX]

Supplementary Table 1: Number of SNPs and their distribution on the chromosomes

| **GGA** | **Length (Mb)** | **Average SNPs (interval in Mb)** | **Number of SNP** | **Max distance between SNPs (Mb)** | **Min distance between SNPs (base)** |
| --- | --- | --- | --- | --- | --- |
| 1 | 195.3 | 0.96 | 3443 | 0.4 | 18 |
| 2 | 148.8 | 0.1 | 2399 | 0.6 | 1 |
| 3 | 110.4 | 0.09 | 1996 | 0.5 | 46 |
| 4 | 90.2 | 0.09 | 1602 | 0.4 | 5 |
| 5 | 59.6 | 0.01 | 976 | 0.5 | 46 |
| 6 | 34.9 | 0.06 | 832 | 0.3 | 16 |
| 7 | 36.2 | 0.08 | 844 | 0.4 | 145 |
| 8 | 28.8 | 0.09 | 650 | 0.4 | 124 |
| 9 | 23.4 | 0.07 | 474 | 0.3 | 772 |
| 10 | 19.9 | 0.05 | 621 | 0.2 | 1 |
| 11 | 19.4 | 0.08 | 648 | 0.7 | 1 |
| 12 | 19.9 | 0.05 | 583 | 0.2 | 3 |
| 13 | 17.8 | 0.05 | 572 | 0.2 | 145 |
| 14 | 15.2 | 0.06 | 456 | 0.2 | 56 |
| 15 | 12.7 | 0.05 | 551 | 0.2 | 94 |
| 16 | 0.535 | 0.01 | 5 | 0.08 | 15142 |
| 17 | 10.4 | 0.05 | 379 | 0.2 | 51 |
| 18 | 11.2 | 0.05 | 358 | 0.2 | 90 |
| 19 | 9.9 | 0.04 | 340 | 0.3 | 6 |
| 20 | 14.3 | 0.04 | 755 | 0.2 | 73 |
| 21 | 6.8 | 0.03 | 359 | 0.1 | 90 |
| 22 | 4.1 | 0.08 | 135 | 0.2 | 1 |
| 23 | 5.7 | 0.04 | 174 | 0.2 | 13 |
| 24 | 6.3 | 0.03 | 254 | 0.1 | 20 |
| 25 | 2.2 | 0.03 | 71 | 0.1 | 20 |
| 26 | 5.3 | 0.03 | 138 | 0.2 | 6 |
| 27 | 5.2 | 0.06 | 99 | 0.5 | 160 |
| 28 | 4.7 | 0.03 | 252 | 0.2 | 12 |
